# Interfacially Reinforced Crosslinked Binder with Structural Integrity for Stable Micro‐Sized Silicon Anodes in All‐solid‐state Batteries

**DOI:** 10.1002/advs.202600022

**Published:** 2026-03-02

**Authors:** Chanho Lee, Yuri Nam, Incheol Jeong, Seo Eun Lee, Taewook Kim, Jinhyung Kim, Wooseup Jo, Moonsu Yoon, Jongkyeong Lim, Seho Sun, Junghyun Choi, Chan Ho Park, Dongsoo Lee

**Affiliations:** ^1^ School of Chemical Biological and Battery Engineering Gachon University Seongnam Republic of Korea; ^2^ Resources Utilization Research Center KIGAM Daejeon Republic of Korea; ^3^ Department of Mechanical Engineering Gachon University Seongnam Republic of Korea; ^4^ School of Chemical Engineering Yeungnam University Gyeongsan Republic of Korea

**Keywords:** all‐solid‐state batteries, binder, hydrogen bonding, in situ crosslinking, micro silicon anodes

## Abstract

All‐solid‐state batteries (ASSBs) have attracted considerable attention as next‐generation energy storage systems owing to their high energy density and safety. However, their performance is critically limited by insufficient solid–solid interfacial contact and severe chemomechanical degradation, particularly for micro‐sized silicon (µSi) anodes that undergo large volume changes during cycling. In this study, we report an interfacially reinforced crosslinked binder (IRCB) designed to stabilize µSi anodes in ASSBs by simultaneously addressing mechanical integrity and interfacial stability. The IRCB is synthesized via a facile crosslinking reaction between 1,4‐butanediol diglycidyl ether and ethylenediamine, forming a robust 3D polymer network. This crosslinked structure enhances mechanical constraint, maintains interparticle contact, and provides ether‐rich domains that facilitate Li^+^ transport, while strong hydrogen bonding improves adhesion to µSi surfaces. As a result, carbon‐free µSi anodes employing IRCB exhibit markedly improved electrochemical stability, delivering 90% capacity retention after 300 cycles at 1 C, compared with only 16% for conventional PVDF‐based electrodes. Structural and interfacial analyses reveal that IRCB effectively mitigates particle displacement and suppresses interfacial degradation with sulfide solid electrolytes. This work demonstrates that rational binder engineering is a key enabler for achieving stable and high‐performance µSi anodes in ASSBs.

## Graphical abstract

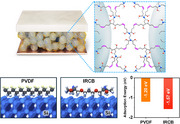

## 1 Introduction

All‐solid‐state batteries (ASSBs) have gained increasing attention as next‐generation energy storage systems due to their superior energy density and enhanced safety compared to conventional Li‐ion batteries (LIBs) employing liquid electrolytes (LEs) [1, 2, 3]. Unlike conventional LIBs, where liquid–solid interfaces facilitate Li‐ion transport, ASSBs critically rely on intimate solid–solid contact, which governs Li‐ion transport efficiency and electrochemical stability [4, 5]. However, achieving intimate solid–solid contact remains challenging due to the chemo‐mechanical failure [6, 7]. Especially, the interfaces between active materials (AMs) and inorganic solid electrolytes (ISEs) play a pivotal role in determining the long‐term cycling performance of ASSBs, primarily due to interfacial side reactions and Li‐ion transport kinetics [8, 9]. Therefore, the selection of appropriate AMs and ISEs, along with the design of mechanically compliant interfaces, is essential to ensure cell‐level reliability in practical ASSB architectures [10].

Among various candidates, micro‐sized silicon (µSi) stands out as a highly promising anode material for ASSBs, offering a high theoretical capacity (3,579 mAh g^–^^1^, Li_3.75_Si), a low redox potential of 0.3 V vs. Li^+^/Li, as well as natural abundance and cost effectiveness [11, 12]. In LIBs, the repeated lithiation and delithiation of Si leads to severe volume expansion and contraction. These substantial volume changes trigger the spontaneous formation of solid electrolyte interphase (SEI) and Li inventory loss, ultimately resulting in poor electrochemical performance [13, 14]. In contrast, the incorporation of ISEs offers the potential to improve interfacial stability and reduce interfacial side reactions due to the limited passivating layer [15]. Tan et al. achieved remarkable cycling stability of carbon‐free µSi anodes with Li_6_PS_5_Cl (LPSCl) as an ISE in ASSBs [16]. The limited 2D interfacial contact between the µSi electrode and the LPSCl layer helps suppress further decomposition of LPSCl at the µSi/LPSCl interface. In contrast, an interconnected 3D interfacial network, where µSi and LPSCl are homogeneously blended in the anode composite, promotes extensive decomposition of LPSCl across the enlarged interfaces, leading to rapid capacity fading [17]. Severe chemo‐mechanical degradation remains a critical challenge for µSi anodes in ASSBs, causing particle fracture, interfacial delamination, and the formation of unstable interphases [18]. The intrinsic volume expansion of Si further exacerbates mechanical disruption, leading to the loss of electronic percolation, internal electrode disconnection, and ultimately irreversible capacity fading [19].

To overcome these limitations, the development of robust polymeric binders is considered one of the most critical strategies for achieving electrode integrity and interfacial stability of µSi anodes during cycling [20]. Although carboxymethyl cellulose (CMC) and styrene‐butadiene rubber (SBR) aqueous binders are extensively used in conventional LIB anodes, they lack sufficient mechanical strength and adhesion to effectively accommodate the substantial volume expansion of µSi anodes [21]. Poly(vinylidene fluoride) (PVDF) is currently used in various studies due to its thermal and chemical stability [16, 17]. However, its weak interaction with µSi and insufficient elasticity hinder effective stress relaxation during repeated lithiation and delithiation [22]. Therefore, conventional binders designed for LIBs remain inadequate for controlling the large volume changes of µSi anodes. Recent studies have reported that the electrochemical performance of µSi anodes can be improved through tailored binder design [23, 24, 25, 26, 27, 28]. In these studies, binders were shown to play an active role beyond simple particle binding, contributing to the maintenance of interfacial contact and mechanical integrity of Si electrodes under repeated volume changes [27]. Moreover, recent binder design strategies have demonstrated that tailoring the chemical structure and interfacial functionality of binders can significantly enhance the cycling stability of µSi anodes, even in carbon‐free electrode configurations [28]. Accordingly, further development of binder systems capable of forming strong chemical interactions with µSi while providing robust mechanical binding and efficient Li^+^ transport remains essential for realizing practical µSi anodes in ASSBs.

In this study, we report a novel interfacially reinforced crosslinked binder (IRCB) designed to address the chemo‐mechanical challenges of µSi anodes in ASSBs. The IRCB is synthesized through a facile in situ crosslinking of 1,4‐butanediol diglycidyl ether (BDDE) and ethylenediamine (EDA), forming a 3D polymer network. This robust crosslinking network not only enhances the mechanical integrity of µSi anodes but also contributes to enhanced Li^+^ conductivity with ether–rich domains. In addition, its strong hydrogen bonding with µSi could improve particle adhesion and mitigate electrode rupture during cycling. With these advantages, µSi anodes incorporating IRCB demonstrated outstanding electrochemical stability with crack‐free morphologies, showing significantly enhanced capacity retention of 90% at 1 C after 300 cycles compared to only 16% for the PVDF counterpart. Through careful analyses, we reveal that the robust, structurally integrated binder network in µSi anodes enhances interfacial stability with the ISE layer by effectively mitigating interparticle displacement. This study highlights the critical role of advanced binder systems in enabling stable, long‐term cycling of µSi anodes in ASSBs.

## 2 Results and Discussion

**FIGURE 1:**
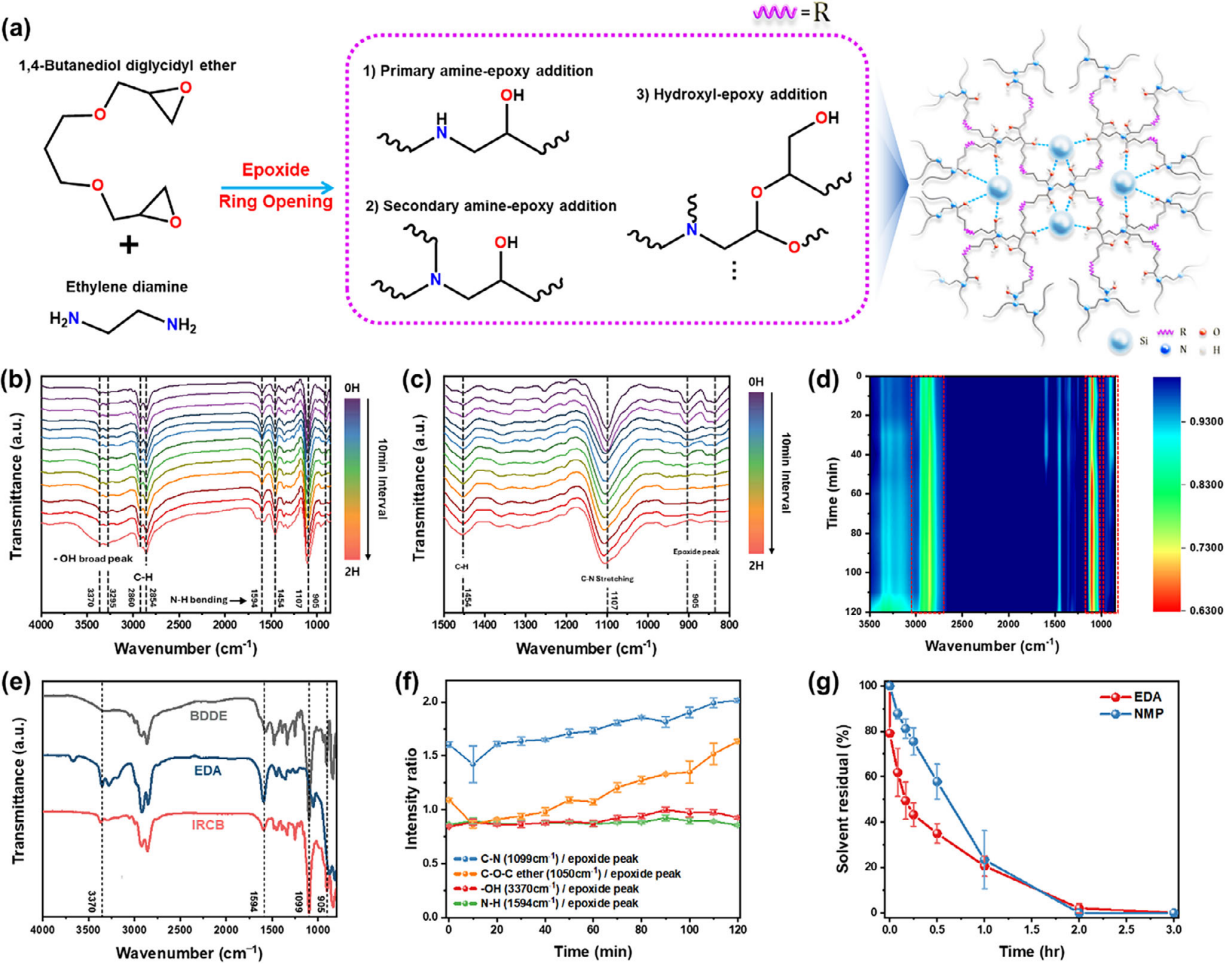
Crosslinking mechanism and spectroscopic validation of the IRCB system. (a) Schematic of epoxide–amine crosslinking between BDDE and EDA (b–d) Time‐resolved FT‐IR spectra and contour map showing the consumption of epoxide groups and formation of C–N and –OH functionalities during curing. (e) Comparative FT‐IR spectra of BDDE, EDA, and the fully cured binder confirming covalent network formation. (f) Normalized intensity ratios of functional groups (–OH, C–N, N–H) to epoxide peak, demonstrating reaction progress. (g) Solvent evaporation behavior of EDA and NMP at 50 °C.

To develop a high‐energy‐density and electrochemically stable binder system for µSi anodes, a 3D polymer network based on epoxide–amine chemistry is proposed (Figure 1a) [29]. In Figure 1a, the “R” groups represent the representative chemical structures formed during the amine–epoxide ring‐opening reactions of BDDE and EDA. The resulting binder architecture, formed through multiple reaction pathways including primary/secondary amine additions and hydroxyl–epoxide reactions, is rich in hydroxyl, amine, and ether linkages [21, 30]. These functional groups not only promote robust 3D hydrogen bonding and covalent anchoring to hydroxylated surfaces of µSi and Cu substrates, but also contribute to enhanced ionic conductivity [31]. In particular, the presence of ether‐rich domains facilitates Li‐ion transport by solvating lithium salts and providing continuous ion migration pathways within the binder matrix [32]. This combination of physical adhesion, chemical bonding, and ion‐conductive functionality results in a binder that is mechanically durable and electrochemically compatible under high‐capacity cycling conditions. In addition to its structural and functional merits, the binder is also fully processable in the liquid phase using BDDE and EDA as precursors. Both components are liquid at room temperature (T_m_ < –60 °C for BDDE and ∼8.5 °C for EDA), allowing direct mixing with µSi powder without the use of conventional organic solvents such as NMP. EDA, used in excess, functions as both a curing agent and reactive diluent, enabling low‐temperature curing (<80 °C) suitable for thermally sensitive electrode systems.

To quantitatively confirm the chemical crosslinking between BDDE and EDA, time‐resolved Fourier‐transform infrared (FT‐IR) spectroscopy was conducted. Figure 1b–d provides a comprehensive overview of the dynamic spectral changes during curing, including representative FT‐IR spectra, peak intensity evolution, and functional group‐specific transitions relevant to the reaction mechanism. Spectra were recorded at 10‐min intervals over a 120‐min curing period. In Figure 1b, c, several key vibrational bands exhibit clear time‐dependent behavior. Notably, the epoxide ring vibration near ∼905 cm^–^^1^ gradually diminished, indicating the progressive consumption of epoxide groups through nucleophilic ring‐opening by amines [33, 34]. Concurrently, the intensity of the C–N stretching band (∼1230 cm^–^^1^) increased, reflecting the formation of covalent bonds between amine and epoxy groups. In addition, the broad –OH stretching band (3300–3500 cm^–^^1^) became more prominent over time. This increase is attributed to the generation of secondary alcohols as a byproduct of epoxide ring‐opening reactions. The N–H bending mode (∼1454 cm^–^^1^) and C–N skeletal vibrations (∼1107 cm^–^^1^) also showed gradual changes, further supporting the continuous crosslinking process [24, 35]. These spectral evolutions are characteristic signatures of the epoxide–amine addition mechanism, involving both primary and secondary amine reactions as well as hydroxyl formation. Based on the near‐complete disappearance of the epoxide‐related peak by 30 min, this duration was selected as the optimal curing time for the binder system in subsequent experiments. Figure 1d presents a contour plot highlighting the temporal evolution of the FT‐IR spectra during the curing process. In this plot, the *x*‐axis represents the wavenumber, the *y*‐axis indicates the reaction time, and the color intensity corresponds to the absorbance magnitude. A distinct absorption band near 910 cm^–^^1^, attributed to the epoxide group of BDDE, exhibits a high initial intensity but progressively diminishes over time. This decrease in peak intensity reflects the continuous consumption of epoxide groups as the crosslinking reaction proceeds. FT‐IR analysis showed that most epoxide groups were consumed within the first 30 min, after which the reaction slowed due to diffusion limitations (Figure S1). Although near‐complete curing occurs by 60–70 min, longer curing significantly increases viscosity and reduces processability. Therefore, 30 min was selected as the practical pre‐curing time, followed by post‐curing to consume remaining reactive groups.

Figure 1e compares the FT‐IR spectra of the individual precursors (pure BDDE and EDA) with that of the fully cured binder, highlighting the chemical transformations that occur upon crosslinking. The spectrum of BDDE exhibits a prominent absorption band at 910 cm^–^^1^, corresponding to the epoxide functional group, while EDA shows characteristic peaks associated with N–H bending and primary amine functionalities. In contrast, the spectrum of the cured binder no longer displays the distinctive peaks of either precursor. Instead, new absorption bands emerge, notably those corresponding to C–N stretching and –OH stretching, indicative of successful epoxide–amine ring‐opening and subsequent polymer network formation. These spectral changes confirm that the binder was formed via covalent crosslinking, rather than physical blending, ensuring the creation of a chemically bonded 3D network. ^1^H NMR and TGA further support the formation of a covalent network (Figures S2 and S3). Figure 1f presents the time‐dependent evolution of key peak intensity ratios, namely, –OH/epoxide, C–N/epoxide, and N–H/epoxide, used to track the progress of the curing reaction [14]. The C–N/epoxide ratio exhibits a gradual increase followed by a plateau, indicating the progressive formation of C–N bonds via nucleophilic attack of amine groups on the epoxide ring and the eventual saturation of reactive sites [36]. The –OH/epoxide and N–H/epoxide ratio demonstrates a more pronounced and continuous increase. This trend supports the mechanism of secondary alcohol formation and residual primary amine bonds due to the use of EDA in excess during epoxide ring opening, consistent with the expected outcome of the curing process. Collectively, the quantitative spectral analyses provide clear evidence that the crosslinking predominantly follows the amine–epoxide addition mechanism. Moreover, employing normalized peak intensity ratios enables real‐time, quantitative monitoring and control of both the extent and kinetics of the curing process with high sensitivity and reliability. Figure S4 summarizes the two‐step curing process, where pre‐curing initiates partial crosslinking and post‐curing completes the reaction throughout the electrode. Figure S5 further confirms interfacial bonding between IRCB and µSi, as evidenced by redshifted Amide bands and broadened O–H/N–H stretching, indicating strengthened hydrogen bonding at the interface. In particular, the simultaneous shift of both Amide A and Amide I bands suggests a reduction in free N–H/O–H groups and the formation of a more strongly coordinated environment around the polymer backbone [37, 38]. In addition to the chemical characterization, process compatibility of the binder system was evaluated to ensure practical applicability in electrode fabrication. Furthermore, EDA exhibits more favorable evaporation characteristics than NMP. As shown in Figure 1g, Figure S6, and Table S1, EDA‐based slurries (80 µm thick on Cu foil) dried at 50 °C and 80 °C exhibit efficient solvent removal while avoiding excessively rapid evaporation, which could otherwise induce film cracking. This balance supports compatibility with existing slurry processing lines and offers potential benefits in terms of manufacturing simplicity and energy efficiency. As summarized in Table S2, EDA also exhibits favorable environmental and safety indicators, including higher biodegradability and lower regulatory burden compared with NMP, reinforcing the advantages of adopting an NMP‐free process.

**FIGURE 2:**
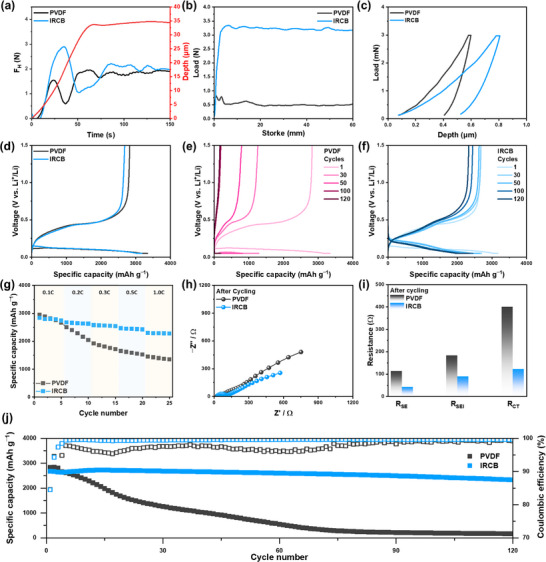
(a) Interlayer shear force profiles obtained from SAICAS measurements at a fixed cutting depth. (b) Tensile stress–strain curves measured using a UTM. (c) Load–displacement curves from nanoindentation measurements. (d) Initial voltage profiles of half cells containing µSi anodes with PVDF and IRCB binders, respectively, at 0.1 C. (e), (f) Voltage profiles of half cells containing µSi anodes with PVDF and IRCB binders during cycling. (g) Rate performance of half cells from 0.1 C to 1.0 C with recovery at 0.2 C. (h) Electrochemical impedance spectra of half cells measured after cycling at 0.2 C. (i) Resistance components (R_SEI_, R_CT_) extracted from impedance spectra after cycling. (j) Cycling performance of half cells with µSi anodes over 120 cycles at 0.2 C.

To quantitatively assess the mechanical reinforcement imparted by the IRCB binder, three distinct evaluation techniques were employed to investigate interfacial shear strength, film‐level adhesion, and local deformation response. Surface and interfacial cutting analysis system (SAICAS) measurements revealed that the IRCB electrode exhibited significantly higher interfacial shear force (F_H_) compared to its PVDF counterpart under a fixed cutting depth of ∼34 µm (Figure 2a) [39]. The IRCB electrode exhibited a stable force plateau in the range of 2.0–2.2 N, while the PVDF electrode showed a delayed and lower F_H_ (∼1.8 N) accompanied by noticeable fluctuations. This result was consistently reproduced across repeated trials (Figures S7 and S8), indicating that the 3D hydrogen bonding and covalent anchoring to hydroxylated surfaces of µSi effectively reinforce robust binding properties in the electrode [40]. In contrast, the PVDF electrode exhibited relatively low and non‐uniform interfacial adhesion, primarily due to the limited chemical bonding associated with functional groups. To evaluate the interfacial adhesion between the electrode and the current collector, 180‐degree peel tests were conducted (Figure 2b). The IRCB electrode exhibited a consistently high peel force of 3.2–3.4 N across the entire testing range, indicating strong and stable adhesion [41]. In contrast, the PVDF‐based electrode showed a substantially lower peel strength, with an initial peak near 0.7 N that rapidly declined below 0.5 N, suggesting poor interfacial integrity and mechanical cohesion. The covalently crosslinked network in the IRCB electrode promotes strong interparticle cohesion and stable interfacial bonding between the electrode and the current collector. This mechanical integrity is critical for preserving interfacial contacts throughout prolonged electrochemical cycling.

To investigate the mechanical properties of the electrode at the microscale, nanoindentation tests were conducted (Figure 2c) [42]. The PVDF electrode showed shallower penetration and higher apparent stiffness, indicating a brittle interfacial nature with limited deformability and poor energy dissipation. In contrast, the IRCB electrode exhibited a more ductile load–depth profile, marked by deeper penetration under equivalent stress. This ductility is not a mechanical weakness but a functional advantage, allowing the binder to accommodate the substantial volume changes of µSi during lithiation and delithiation [43]. This fact is further confirmed by differential scanning calorimetry (Figure S9) and dynamic mechanical analysis (Figure S10). The relatively low glass transition temperature (T_g_) value ​​(18°C by DSC and 16°C by DMA) is attributed to the characteristics of intrinsic properties of building blocks (EDA, BDDE, and their derivatives), while IRCB exhibits stable modulus and damping properties even after T_g_ owing to the 3D‐network (storage modulus of approximately 11.8 MPa and a damping factor (tan δ) of 0.36 at 25°C). This combination of moderate stiffness and high damping indicates a viscoelastic behavior, in which the binder preserves mechanical integrity while efficiently dissipating applied stress. Such viscoelastic characteristics are particularly beneficial for accommodating the repeated volume expansion and contraction of active materials during electrochemical cycling. As a result, it is beneficial to mitigate crack formation and propagation, and interfacial delamination. On the other hand, the insufficient mechanical strength of PVDF promotes localized stress accumulation, which ultimately compromises binder integrity and long‐term structural stability.

Prior to evaluating the electrochemical performance of the IRCB system, a baseline comparison was conducted using conventional binders such as CMC/SBR, aqueous poly(acrylic acid) (PAA), and PVDF under different stack pressures (30 and 50 MPa). The µSi and LPSCl used in this study showed high crystallinity and well‐defined crystallographic structures (Figure S11) [44]. The electrodes prepared with commercial binders were evaluated under identical conditions (Figure S12). PVDF exhibited the most stable electrochemical behavior and was selected as the benchmark for comparison in this study. As CNT additives accelerated LPSCl decomposition and degraded cycling stability, all subsequent electrodes were fabricated without conductive carbon (Figure S13) [16].

To investigate the electrochemical properties of IRCB, µSi anodes were prepared using the same electrode composition with an areal capacity of 3 mAh cm^–^^2^. As shown in Figure 2d, the initial discharge/charge profiles of the PVDF and IRCB electrodes are directly compared. The PVDF electrode exhibited an initial discharge/charge capacity of 3340/2825 mAh g^–^^1^ with an initial Coulombic efficiency (ICE) of 84.6%. Similarly, the IRCB electrode delivered 3172/2680 mAh g–^1^ with an ICE of 84.5%. These results confirm that the IRCB binder does not induce additional parasitic reactions such as excessive SEI formation and maintains stable interfacial compatibility with LPSCl (Figure 2e, f). The rate capability highlights a clear difference in electrochemical performance between the two systems (Figure 2g). At a low current density of 0.1 C, both electrodes exhibited comparable capacities. However, as the current density increased, the PVDF electrode exhibited poor rate performance, showing a rate retention of 49% at 1.0 C relative to 0.1 C. The limited rate capability is attributed to sluggish Li^+^ kinetics and insufficient mechanical integrity of the PVDF binder. In contrast, the IRCB electrode demonstrated superior rate capability, achieving a rate retention of 75% (1.0 C/0.1 C). These findings suggest that the IRCB binder facilitates enhanced Li^+^ kinetics while maintaining structural integrity and interfacial stability, enabling consistent performance under high current densities. The voltage profiles and the differential capacity (dQ/dV) plots further substantiate that the PVDF electrode exhibits increased polarization and flattened plateaus at higher C‐rates, indicative of sluggish ion transport and increased overpotential (Figure S14). In contrast, the IRCB electrode maintains sharp and distinct voltage plateaus even at 1.0 C, consistent with improved kinetic response under high‐rate conditions.

To investigate the origin of the performance disparity, electrical resistance measurements were conducted using a HIOKI impedance analyzer (Figure S15). The composite volume resistivity of the electrode refers to the bulk electrical resistance of the electrode film, which includes both µSi and the binder [45]. In contrast, the interface resistance represents the contact resistance between the electrode and the current collector, characterizing the efficiency of electron transfer across their interface [46]. The IRCB electrodes exhibited lower composite volume resistivity and interface resistance than the PVDF electrodes. The IRCB more effectively sustains conductive pathways with ether–rich domains, thereby contributing to the superior rate performance observed in Figure 2g. The electrochemical impedance spectroscopy (EIS) data before cycling proved that the PVDF electrode exhibited larger semicircles (charge transfer resistance, R_CT_) compared to those of the IRCB electrode before cycling (Figure S16). EIS measurements after cycling clearly demonstrate distinct differences in interfacial degradation between PVDF and IRCB electrodes. (Figure 2h, i). The PVDF electrode shows substantially higher R_SEI_ and R_CT_ values, reflecting pronounced interfacial degradation during repeated cycling [47]. In contrast, the IRCB electrode maintained stable interfaces with a slight increase, confirming its effectiveness in suppressing impedance growth during extended cycling.

The cycling performance at 0.2 C further highlights the influence of binder chemistry for µSi anodes in ASSBs (Figure 2j). The PVDF electrode exhibited continuous capacity fading, and its capacity retention was only 7% after 120 cycles. The poor cycle performance of the PVDF electrode is attributed to cumulative mechanical fatigue and loss of interfacial contacts. The unstable physicochemical and structural stability of PVDF cannot withstand the volume changes of µSi during cycling. In stark contrast, the IRCB electrode exhibited excellent cycle performance with a capacity retention of 92.1% over 120 cycles. The result is attributed to the structural and interfacial stability, and the fast Li^+^ kinetics associated with the unique 3D network structure and multiple functional groups of IRCB. The corresponding Coulombic efficiency (CE) also supports this trend. The PVDF electrode showed lower and fluctuating CEs during cycling, whereas the IRCB electrode maintained a stable CE over 99.7%, indicating a reversible electrochemical reaction. The dQ/dV plots further validate the reversible capacity utilization of the IRCB electrode, showing consistent redox peaks (Figure S17).

**FIGURE 3:**
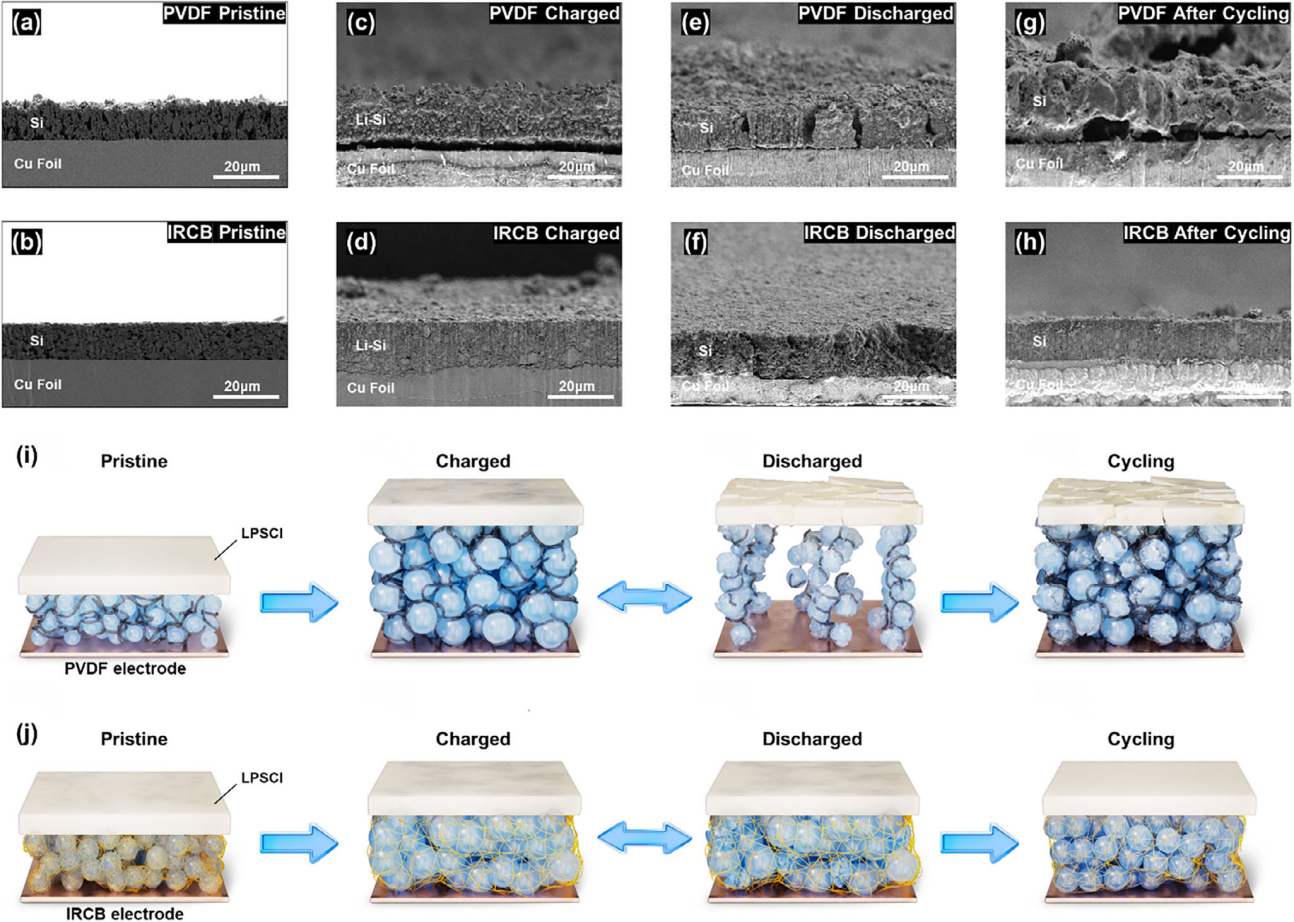
Comprehensive microstructural evolution of µSi electrodes during cycling. (a–h) Cross‐sectional SEM images of PVDF and IRCB electrodes in the pristine state (a, b), after the first charge (c, d), after the first discharge (e, f), and after cycling (g, h). (i), (j) Schematic illustrations showing the structural evolution during cycling for PVDF (i) and IRCB (j) electrodes.

Figure 3 presents the comprehensive microstructural evolution of µSi electrodes during cycling. In the pristine state, both electrodes exhibit densely packed microstructures (Figure 3a, b). Upon charging, both electrodes showed volume expansion due to the alloying reaction with Li, forming Li*_x_* Si phases (Figure 3c, d) [48]. The PVDF electrode exhibited significant structural degradation and pronounced necking between µSi particles, attributed to electrochemical sintering [49]. In contrast, the IRCB electrode maintained morphological stability with reduced necking owing to its 3D covalently crosslinked binder network, which effectively suppresses interparticle fusion and mechanical coalescence. During discharging, the electrodes showed clear differences in structural stability (Figure 3e, f). The PVDF electrode exhibited vertical cracks with columnar structure attributed to the nonuniform internal stress dissipation caused by accumulated mechanical stress from electrochemical sintering [17]. The formation of microcracks leads to contact loss within the electrode, further exacerbating the electrochemical performance of ASSBs [50]. In contrast, the IRCB electrode maintains a densely packed microstructure. IRCB with the 3D crosslinked network architecture efficiently accommodates volumetric fluctuations by mitigating internal stress accumulation during charging and promoting facile stress relaxation during discharging. After electrochemical cycling, the cumulative impact of mechanical stress became even more pronounced (Figure 3g, h). The PVDF electrode showed severe structural degradation, including microcracks, contact loss, and interfacial degradation due to the repetitive inhomogeneous stress accumulation and relaxation. In contrast, the IRCB electrode maintains its morphological integrity, with densely packed µSi domains and no apparent mechanical failure. These observations are further illustrated by the schematic illustration (Figure 3i,j)

**FIGURE 4:**
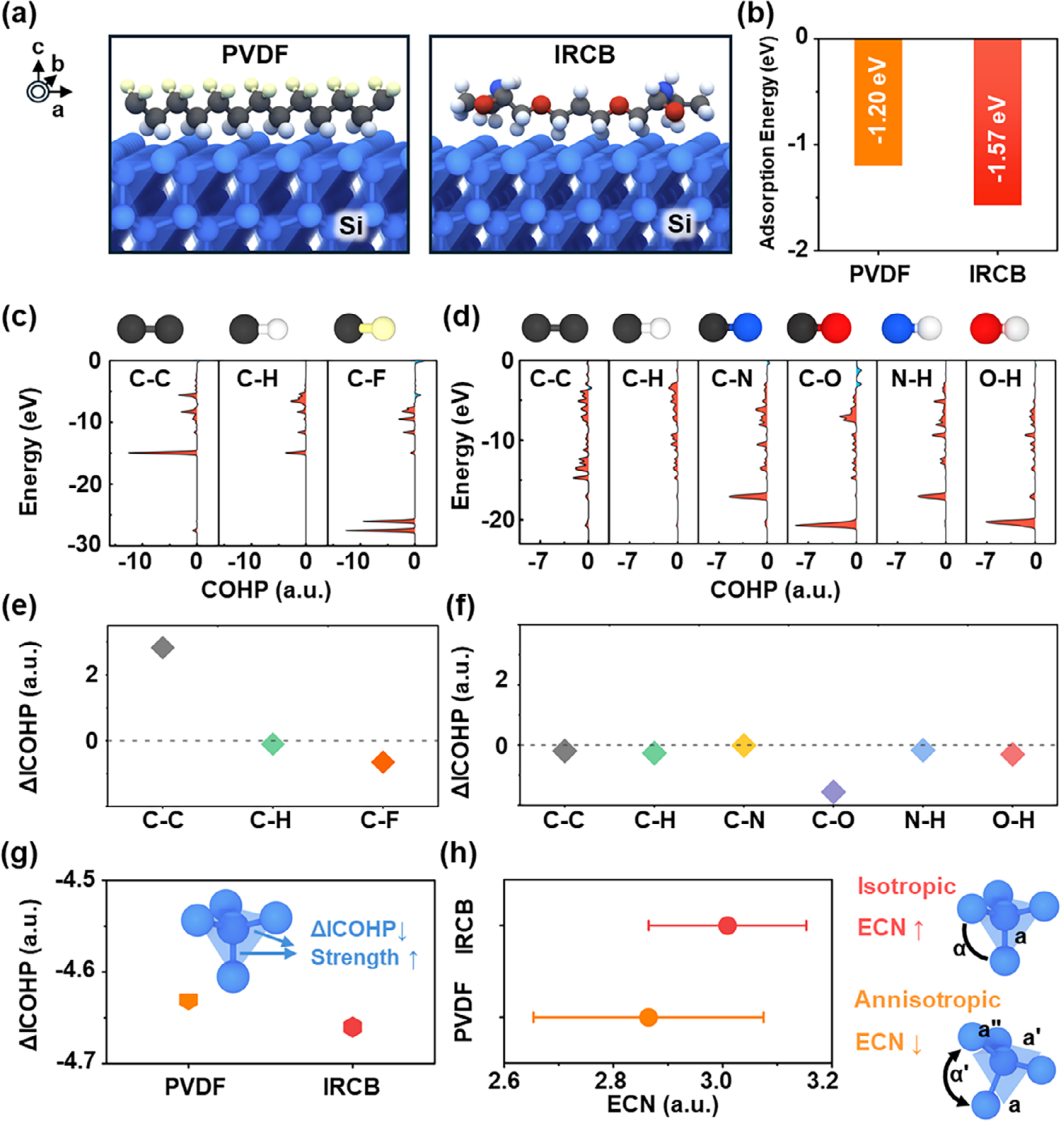
(a) Schematic illustration of PVDF and IRCB adsorption on the Si (111) plane. (b) Adsorption energies of PVDF and IRCB. COHP profiles of elemental bond in (c) PVDF and (d) IRCB on Si (111). ΔICOHP of elemental bond in (e) PVDF and (f) IRCB. (g) ΔICOHP of Si‐Si bond with binders. (h) Average ECN of Si with PVDF and IRCB.

Atomistic simulations were performed using DFT calculations to elucidate the underlying mechanisms behind the enhanced electrochemical performance and structural integrity of µSi anodes prepared with IRCB. To ensure electrode integrity and uniform dispersion of the components, binders should provide mechanical and chemical reliability, including appreciable interfacial adhesion with the active material and inherent chemical robustness. Otherwise, weak interaction forces between the binders and the active material lead to interface failure, or the chemical decomposition of the binders themselves can cause degradation failure [51, 52]. Therefore, the adsorption characteristics and chemical stability of the binders are explored. Figure 4a presents a schematic illustration of the adsorption of PVDF and IRCB binders on Si. A Si (111) plane slab, which is a closely packed and stable configuration, was employed for the investigation. Figure 4b shows the adsorption energy of binders on Si. PVDF and IRCB exhibited adsorption energies of –1.20 and –1.56 eV, respectively. The lower adsorption energy of IRCB suggests improved adsorption properties and suppression of delamination. We further performed adsorption energy calculations on amorphous Si (Figure S18a) and Li_x_Si (Figure S18b). Figure S18c, d present the radial distribution functions, which validate the crystallinity of the crystalline Si structure and the amorphous Si structure, respectively. While the crystalline Si structure exhibits discrete peaks, which is a characteristic of a regularly repeating atomic array, the amorphous Si structure shows peak broadening, indicating successful representation of amorphization. Figure S18e, f displays the adsorption energies of PVDF and IRCB on amorphous Li and amorphous Li_x_Si, respectively. IRCB still exhibits stronger adsorption under both conditions compared to PVDF, indicating that IRCB would maintain good adhesion to Si during charging. Figure 4c, d display the crystal orbital Hamilton population (COHP) profiles of elemental bonds in PVDF and IRCB, respectively, after adsorption on the crystalline Si surface. Figure 4e, f summarizes the differences in integrated COHP (ICOHP) values between before and after adsorption. Positive ΔICOHP values imply the destabilization of bonding, whereas negative values indicate stabilization. The ΔICOHP values for C‐C, C‐H, and C‐F bonds in PVDF were 2.83, –0.10, and –0.65, respectively, while those for C‐C, C‐H, C‐N, C‐O, N‐H, and O‐H bonds in IRCB were –0.19, –0.26, –0.01, –1.56, –0.17, and –0.31, respectively. Unlike most bonds, the C‐C bond in PVDF exhibited severe destabilization after adsorption, which is attributed to the growth of anti‐bonding characteristics at the expense of bonding interactions. The robust bonding framework of IRCB contributes to its chemical stability and maintains its crosslinking structural integrity upon stress during cycling. Figure 4g shows the ΔICOHP values of surface Si‐Si bonds upon binder adsorption. The ΔICOHP value of surface Si atoms upon PVDF adsorption (–4.63) was higher than that with IRCB (–4.66), suggesting that Si bonding is more stable with IRCB. Figure 4h presents the effective coordination number (ECN) of Si in the crystal structure depending on the adsorbed binders. The average ECN of Si with IRCB (3.01) was higher than with PVDF (2.86). A higher ECN indicates more isotropic bonding within the lattice and higher crystal symmetry. Therefore, the structural frustration and amorphization of Si could be mitigated, thereby enhancing structural integrity when using IRCB. For future exploration, a systematic investigation varying the adsorption energy and analyzing structural integrity would offer a new perspective. Furthermore, simulations of elastic properties beyond the atomic scale could provide additional insights.

**FIGURE 5:**
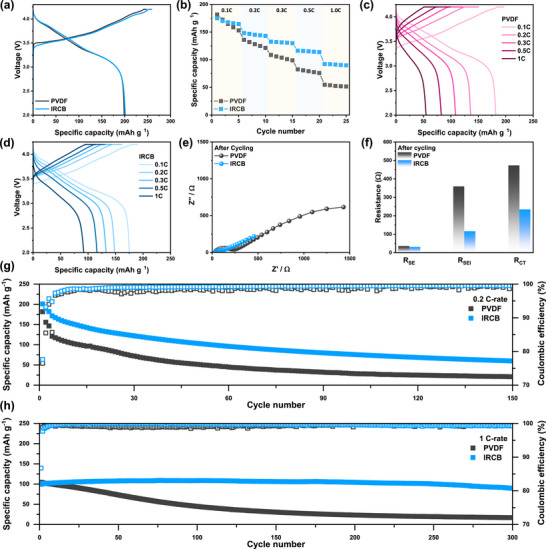
(a) Initial cycle voltage profiles at 0.1 C for full cells with µSi electrodes containing PVDF or IRCB binders. (b) Rate performance of full cells evaluated from 0.1 C to 1.0 C, followed by capacity recovery at 0.2 C. (c), (d) Voltage profiles of PVDF and IRCB full cells under different C‐rates. (e) Electrochemical impedance spectra of full cells measured after cycling at 0.2 C. (f) Resistance components (R_SEI_, R_CT_) of full cells measured after cycling at 0.2 C. (g) Cycling performance of full cells over 150 cycles at 0.2 C. (h) Cycling performance of full cells over 300 cycles at 1.0 C.

To demonstrate practical feasibility, full cell evaluations were conducted using LiNi_0.8_Co_0.1_Mn_0.1_O_2_ (NCM811) as the cathode in a µSi ‖ LPSCl ‖ NCM811 configuration (Figure 5). Both PVDF and IRCB full cells operated under the external stack pressure of 50 MPa within the voltage range of 2.0–4.2 V. The initial charge/discharge capacities of the PVDF and IRCB cells were 247.5/198.7  and 258.9/201.0 mAh g^–^^1^, respectively (Figure 5a). As the current density increased, a clear divergence in rate capability emerged. The PVDF cell exhibited continuous capacity fading starting from 0.2 C and poor rate capability with a rate retention of 32% at 1.0 C relative to 0.1 C (Figure 5b). In contrast, the IRCB cell demonstrated significantly improved performance, achieving a rate retention of 54% under the same conditions (1.0 C/0.1 C). The voltage profiles and dQ/dV plots at different current densities further clarify the outstanding rate capability of the IRCB cell (Figure 5c, d; Figure S19). The PVDF cell showed large overpotentials and diminished redox peaks upon increasing current densities due to the sluggish Li^+^ transport. However, the IRCB cell exhibited relatively low overpotentials and clear redox peaks even at 1C. This enhanced rate capability of the IRCB cell originates from its intrinsic advantages associated with fast Li^+^ kinetics and facile stress relaxation effect. The EIS analysis of full cells was conducted after cycling to observe interfacial stability and associated resistance evolution (Figure 5e). The EIS data measured before cycling are provided in Figure S20. The PVDF cell exhibited significant resistance evolution, showing higher R_SEI_ and R_CT_ values of 359.3 and 473.3 Ω, respectively, after cycling (Figure 5f). However, the IRCB cell showed lower R_SEI_ and R_CT_ of 116.9 and 235.1 Ω, respectively. These findings further substantiate that the outstanding interfacial stability of IRCB effectively mitigates interfacial impedance evolution during repeated cycling [53].

The cycling performance further validates the structural and electrochemical stability of IRCB (Figure 5g). The PVDF cell exhibited rapid capacity fading during earlier formation cycles and poor cycling stability at 0.2 C over 150 cycles (Figure S21). In contrast, the IRCB cell exhibited stable cycle performance over 150 cycles. The average CEs of the PVDF and IRCB cells were 98.7% and 99.3%, respectively, indicating more reversible lithium utilization and improved interfacial stability in the IRCB anode. These results underscore the structural advantages of the IRCB binder in achieving long‐term cycling stability. To further challenge the mechanical and interfacial resilience of each system, full cell cycling was conducted at 1.0 C (Figure 5h). The PVDF cell showed continuous capacity fading, and its capacity retention was 16% after 300 cycles. In contrast, the IRCB cell exhibited excellent cycle performance with a capacity retention of 90% after 300 cycles. The voltage profiles and dQ/dV plots further prove the electrochemical stability of the IRCB cell (Figure S22). At higher C‐rates, the increased overpotentials at both the cathode and anode limit the effective lithiation depth of Si, resulting in lower capacity utilization and, consequently, reduced volume changes and mechanical strain accumulation (Figure S24). Hence, the higher capacity retention observed at 1 C is attributed to the lower capacity utilization compared with 0.2 C.

Full cell evaluations at reduced stack pressures of 10 and 5 MPa (Figures S24 and S25) confirm the pressure‐tolerant behavior of the IRCB electrode. The PVDF cell exhibited severe polarization and rapid capacity fading due to loss of interfacial contact under low pressure. In contrast, the IRCB cell maintained low overpotentials and stable cycling, attributed to the mechanically compliant, crosslinked binder network that preserves solid–solid contact and suppresses interfacial void formation. The initial discharge/charge capacities at 5 MPa were 125.8/236.1 mAh g^–^^1^ (ICE 53.3%) for PVDF and 171.5/238.7 mAh g^–^^1^ (ICE 71.9%) for IRCB. GITT results at 5 MPa (Figure S26) further demonstrate that the IRCB electrode provides higher Li^+^ diffusion coefficients than PVDF, consistent with the smaller impedance growth observed in EIS and DRT results. These results verify that IRCB maintains interfacial stability and efficient ion transport even under the low stack pressure. Furthermore, to validate the scalability of the electrode design, high‐loaded full cells with an areal capacity of 6 mAh cm^–^^2^ were evaluated under identical stack pressure conditions (Figure S27). Even under such practical loading, the IRCB cell maintained stable cycling with minimal polarization, while the PVDF cell exhibited rapid capacity fading. In addition, the PVDF electrode shows severe delamination, whereas the IRCB electrode remained intact, confirming superior adhesion and structural integrity (Figure S28).

**FIGURE 6:**
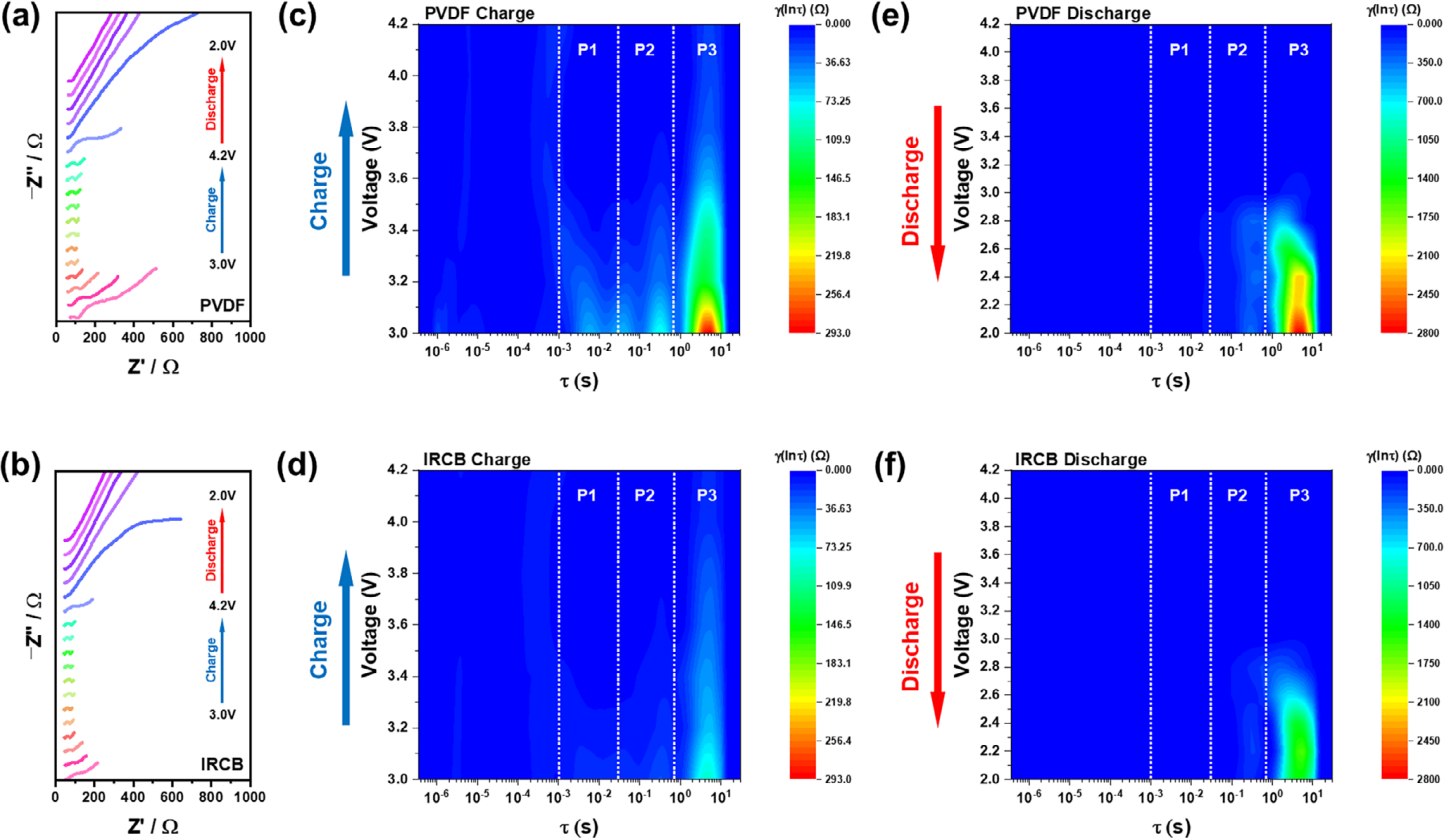
(a), (b) Nyquist plots of full cells with PVDF and IRCB µSi electrodes, measured at multiple state‐of‐charge (SOC) and depth‐of‐discharge (DOD) points during the first cycle. DRT spectra of full cells during charging for PVDF (c) and IRCB (d) electrodes. DRT spectra of full cells during discharging for PVDF (e) and IRCB (f) electrodes. All DRT spectra are resolved into three relaxation regimes (P1–P3) to visualize the evolution of interfacial and transport‐related resistance components over the charge–discharge process.

EIS spectra were collected at multiple state‐of‐charge (SOC) and depth‐of‐discharge (DOD) points during the first charge–discharge cycle, enabling a voltage‐resolved comparison of interfacial behavior of µSi anodes as a function of binder chemistry. During charging, both cells exhibited the highest impedance at 3.0 V, corresponding to the onset of electrochemical activation (Figure 6a). The PVDF cell showed a large semicircle at this voltage, indicative of significant interfacial polarization. Although impedance decreased with increasing voltage, it remained substantially higher than that of the IRCB cell throughout the charging window (Figure 6b). In contrast, the IRCB cell displayed a smaller semicircle at 3.0 V and consistently lower impedance across all voltages, suggesting suppressed R_SEI_ from the early stages of lithiation. During discharge, the Nyquist plots of both cells appeared qualitatively similar, with only moderate impedance increases below 2.8 V. However, these visual similarities masked underlying differences in resistive component evolution, underscoring the limited resolution of conventional EIS in deconvoluting interfacial processes.

To gain deeper insight into the dynamic evolution of interfacial resistance during cycling, distribution of relaxation times (DRT) analysis was performed (Figure 6c–f; Figure S29). By deconvoluting overlapping electrochemical processes in EIS spectra into distinct components based on their characteristic relaxation times (τ), this method provides superior resolution and interpretive accuracy compared to conventional equivalent circuit fitting [18, 54]. The first peak (P1) region is attributed to R_SEI_ at the Si/LPSCl interface. This component is associated with ion transport across decomposition layers formed at the electrode–electrolyte interface and is sensitive to the formation and growth of interfacial byproducts. The second peak (P2) corresponds to R_CT_, reflecting the electrochemical kinetics at the active interface [18]. The third peak (P3) represents long‐range lithium‐ion diffusion resistance within the Si electrode structure, which becomes increasingly significant as lithiation‐induced volume expansion disrupts percolation pathways and induces microstructural stress.

The PVDF cell exhibited notably higher resistance responses in the P1–P3 regions, suggesting the early development of interfacial instability, SEI accumulation, and hindered Li^+^ transport. In contrast, the IRCB cell exhibited consistently low and stable resistance values in these regions. This behavior indicates that the covalently crosslinked binder effectively accommodates the mechanical stress associated with volume changes of µSi while maintaining continuous ionic transport throughout the electrode. Notably, R_SEI_ and R_CT_ were significantly suppressed, confirming the stabilizing effect of the binder on both mechanical and electrochemical performance during initial cycling. To further validate the Li^+^ transport characteristics inferred from DRT analysis, lithium‐ion diffusion behavior was evaluated using the galvanostatic intermittent titration technique (GITT) (Figure S30). During lithiation, both PVDF and IRCB electrodes exhibited comparable diffusivity. In contrast, delithiation revealed a marked divergence, where the PVDF electrode showed significantly reduced diffusivity due to the contact loss while IRCB sustained higher values across the overall DOD regions [55]. This behavior is consistent with the DRT results. These findings confirm that the IRCB binder effectively preserves fast Li^+^ transport with percolated pathways during cycling.

To complement these electrochemical observations with chemical‐level evidence of interfacial bonding, Figure S31 presents the FT‐IR spectra of µSi, IRCB, and the µSi+IRCB composite. Upon composite formation, characteristic vibrational bands of IRCB exhibit clear shifts and broadening, indicating interfacial interactions between surface –OH groups on µSi and the –NH/–OH functionalities of the binder. These changes reflect the formation of hydrogen‐bonded and partially covalent networks during the BDDE–EDA crosslinking reaction. The resulting chemical coupling enhances mechanical cohesion and maintains intimate solid–solid contact, consistent with the reduced interfacial impedance observed in EIS and DRT analyses. Overall, the FT‐IR evidence supports that the multifunctional groups in IRCB strengthen interfacial adhesion and facilitate Li^+^ transport within the µSi composite.

**FIGURE 7:**
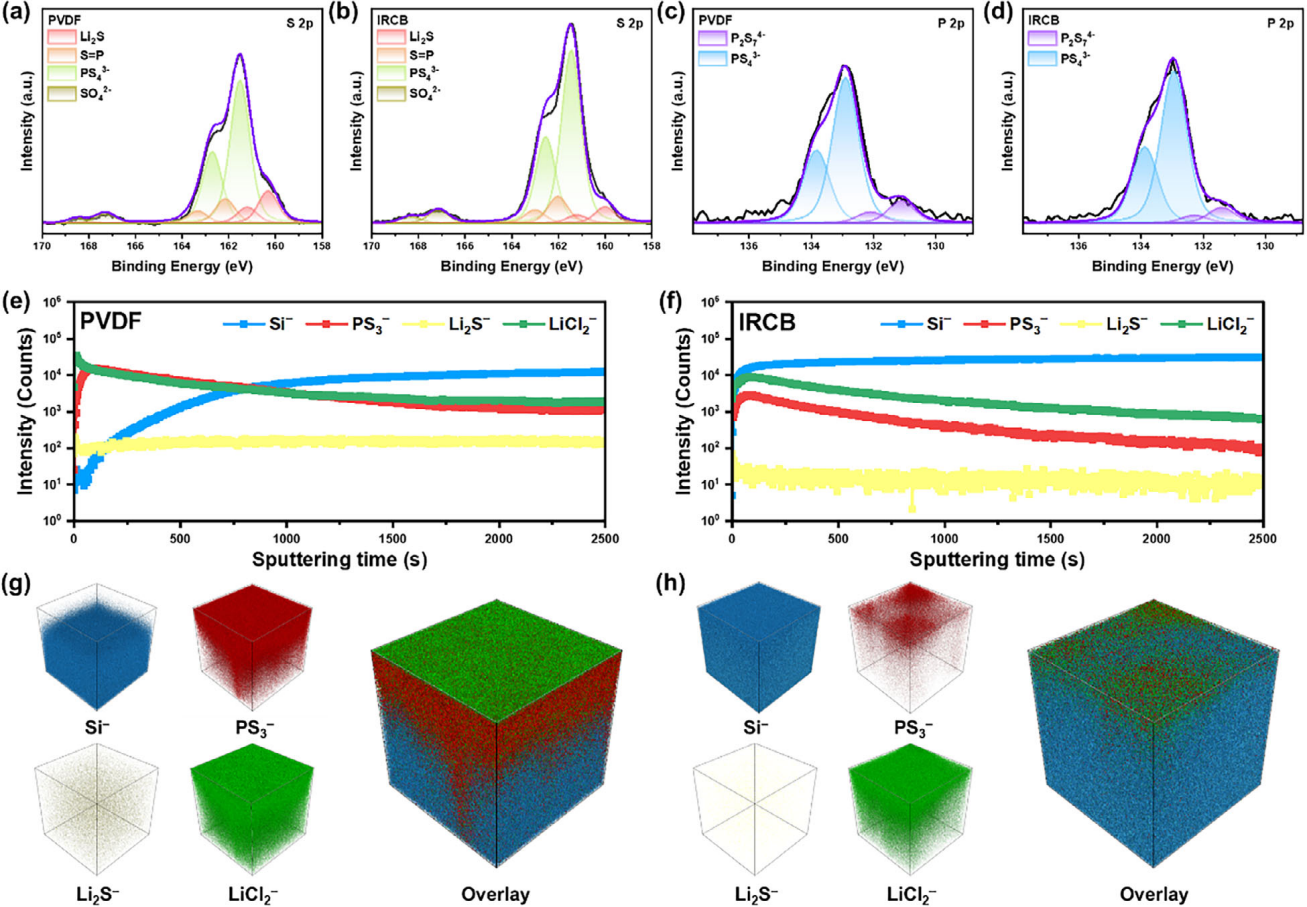
XPS and ToF‐SIMS analysis of PVDF and IRCB electrodes after cycling. (a), (b) S 2p and P 2p XPS spectra of the PVDF electrode. (c), (d) S 2p and P 2p XPS spectra of the IRCB electrode. (e), (f) ToF‐SIMS depth profiles showing the distribution of Si^–^, PS_3_^–^, Li_2_S^–^, and LiCl_2_^–^ fragments. (g), (h) Corresponding 3D ToF‐SIMS renderings of the PVDF and IRCB electrodes.

To investigate the interphase evolution of µSi anodes, postmortem X‐ray photoelectron spectroscopy (XPS) was performed on PVDF and IRCB electrodes after 30 cycles, focusing on the decomposition behavior of LPSCl. In the S 2p spectra, the PS_4_^3–^ peak (∼161.5 eV) corresponds to the intact thiophosphate framework of LPSCl, whereas the P–S–P signal (∼162.2 eV) indicates partial degradation, including oxidized sulfur species and the bridging sulfur species formed via bond cleavage of LPSCl (Figure 7a, b). The distinct Li_2_S peak (∼160.3 eV) reflects the formation of insulating sulfide species from reductive decomposition. The SO_4_^2–^ peak (∼167.3 eV) corresponds to oxidative byproducts generated through side reactions at the µSi/LPSCl interface. Although both electrodes retained the main PS_4_^3–^ signal, the PVDF electrode showed relatively higher Li_2_S intensity, suggesting more extensive reductive decomposition that could increase interfacial resistance. The P 2p spectra further support this trend (Figure 7c, d). The PS_4_^3–^ peak (∼131.1 eV) confirms the preservation of the LPSCl structure, whereas the P_2_S_7_^4–^ peak (∼132.9 eV) indicates decomposition of the thiophosphate framework. The P_2_S_7_^4–^ signal intensity was slightly higher in the PVDF electrode compared to the IRCB electrode, and the PVDF electrode exhibited relatively higher intensities for decomposition‐related peaks in both the S 2p and P 2p spectra. These results suggest that the covalently crosslinked binder in the IRCB electrode could suppress interfacial decomposition, resulting in interfacial stability.

**FIGURE 8:**
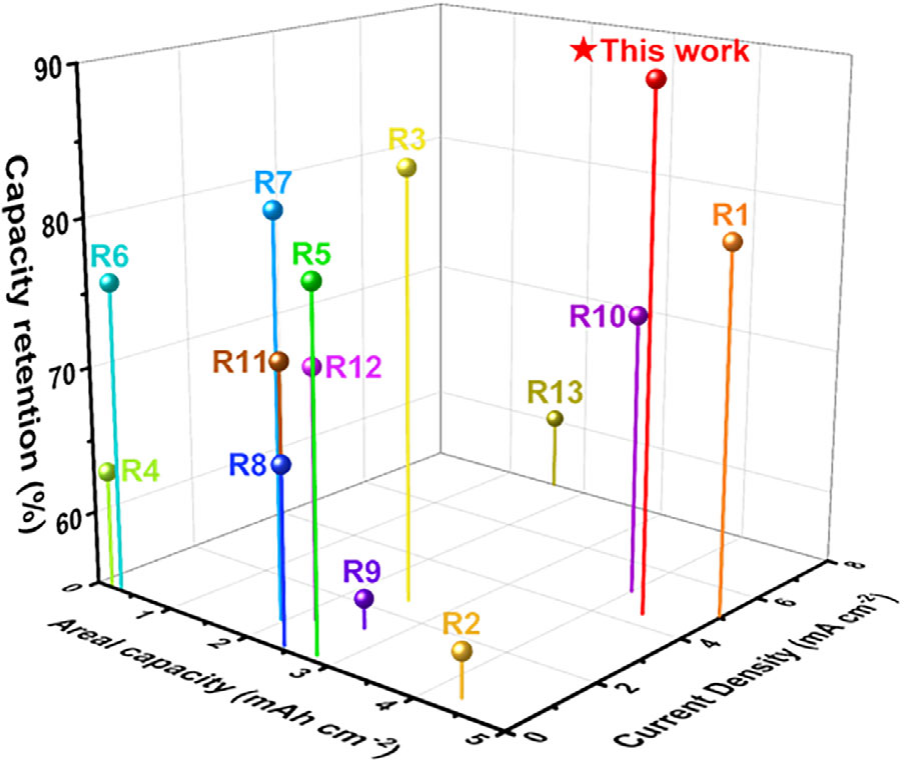
Comparison of cycling performance with full cells employing Si anodes in ASSBs. The benchmarking parameters are summarized in Table S3.

To further investigate the interphase species and their spatial distribution of µSi anodes, time of flight secondary ion mass spectrometry (ToF‐SIMS) was employed. LPSCl undergoes reductive decomposition due to its electrochemical incompatibility with Si. The 2D interfacial contact between the µSi electrode and the LPSCl layer can minimize the interphase evolution [16]. However, the substantial volume fluctuations of Si during cycling induce particle rupture, microcracks, and interparticle displacement. These morphological degradations expose fresh Si surfaces to LPSCl, accelerating interphase growth and promoting the accumulation of decomposition byproducts. For the PVDF electrode, high intensity signals of decomposition products such as PS_3_^–^, Li_2_S^–^, and LiCl_2_^–^ fragments, along with a low intensity signal of Si^–^ fragment, were observed at the µSi/LPSCl interface (Figure 7e) [56]. As sputtering time increased, the signals associated with LPSCl decomposition diminished, while the Si^–^ fragment signal reached saturation. These results indicate that the PVDF electrode undergoes severe chemo‐mechanical degradation at the µSi/LPSCl interface due to the structural collapse. In contrast, the IRCB electrode exhibited relatively lower intensity signals of decomposition byproducts and an already saturated Si^–^ fragment signal near the µSi/LPSCl interface, which substantiates its interfacial stability with structural robustness (Figure 7f). These findings further highlight the advantages of the covalently crosslinked network in the IRCB electrode, which facilitates stress relaxation in µSi and suppresses interparticle displacement and microcracks. The 3D ToF‐SIMS depth profiles demonstrate a pronounced contrast in interphase evolution between PVDF and IRCB electrodes (Figure 7g, h; Figure S32). Severe interphase growth in the PVDF electrode correlates with structural collapse, whereas the IRCB electrode demonstrates minimized interphase and maintains structural integrity, consistent with its ability to accommodate volume changes and suppress electrochemical sintering. As a result, the enhanced interfacial stability of the IRCB electrode leads to outstanding electrochemical performance, as evidenced by the benchmarking results in Figure 8.

## 3 Conclusions

In this study, we developed IRCB via an in situ crosslinking reaction between BDDE and EDA to address the chemo‐mechanical degradation of µSi anodes in ASSBs. The resulting 3D covalently crosslinked polymeric network reinforced interparticle cohesion, mitigated interparticle displacement and microcracks, and preserved electrode integrity under repeated volumetric fluctuations. In addition, the interconnected binder architecture with ether‐rich functional groups provided continuous Li^+^ conducting pathways with uniform interfacial contacts, resulting in reduced interfacial resistance and enhanced Li^+^ diffusivity. These synergistic effects enabled exceptional cycling stability of the high capacity retention of 90% at 1 C over 300 cycles with crack‐free electrode morphologies, as well as the outstanding rate capability in ASSBs. In addition, the robust binder network in µSi anodes incorporating IRCB was beneficial for structural integrity by suppressing microcracks, contact loss, and interfacial degradation. Furthermore, the IRCB electrode delivered enhanced cycling performance even under a reduced stack pressure of 5 MPa, demonstrating its mechanical and interfacial resilience. ToF‐SIMS depth profiling further revealed that while the PVDF electrode exhibited a ruptured and heterogeneous interphase, the IRCB electrode preserved a stable and continuous 2D interphase, confirming its outstanding interfacial stability. This work demonstrates that rational binder design, integrating mechanical robustness, ionic conductivity, and interfacial stability, offers a viable pathway toward the practical realization of high‐energy‐density ASSBs.

## 4 Experimental Section

### 4.1 Materials

µSi powder (Thermo Fisher Scientific, APS 1–5 µm, 99.9%) was used as the active material. The binders were PVDF (Sigma–Aldrich) and the IRCB, which was synthesized in‐house through an in situ crosslinking reaction between BDDE (Sigma–Aldrich) and EDA (Sigma–Aldrich). NMP (Sigma–Aldrich) was used as the solvent for PVDF slurry, while EDA served as both the crosslinker and dispersion medium in the IRCB formulation. The solid electrolyte LPSCl (POSCO JK Solid Solution) was handled entirely in an Ar‐filled glovebox (H_2_O/O_2_< 0.1 ppm) without any further pretreatment. The cathode was composed of NCM811 (Welcos), Super P (C65, Imerys), and polytetrafluoroethylene (PTFE, Daikin), and lithium metal foil (Honjo Metal) was used as the counter electrode in half‐cell tests.

### 4.2 Synthesis of IRCB

IRCB was synthesized via an in situ epoxy ring‐opening reaction between BDDE and EDA. BDDE (183.9 mL/mol) and EDA (66.9 mL/mol) were mixed with µSi using a planetary centrifugal mixer (Thinky ARE‐310) for 10 min. The mixture was then thermally cured at 50 °C for 30 min to initiate crosslinking, followed by two additional mixing steps (10 min each) to ensure homogeneity. No separate solvent was used, as EDA functioned as both the nucleophile and the dispersion medium. Fourier‐transform infrared (FT‐IR) spectra were acquired using a Vertex 70 spectrometer (Bruker) equipped with an attenuated total reflectance (ATR) accessory. A precursor mixture of ethylenediamine and 1,4‐butanediol diglycidyl ether (1:1 molar ratio) was prepared and thermally cured in a convection oven at 50 °C for durations ranging from 0 to 120 min in 10 min intervals. After each curing interval, the sample was immediately placed on the ATR crystal for measurement. Spectra were collected over the range of 4000–800 cm^–^^1^ with a resolution of 4 cm^–^^1^ and 64 scans per sample. Characteristic vibrational modes were analyzed to monitor chemical changes in the binder with respect to curing time.

### 4.3 Material Characterization

Interlayer shear strength was measured using a SAICAS system (NN Type, Daipla Wintec), and film adhesion was evaluated via 180° peel testing using a universal testing machine (UTM). Electrical resistance was analyzed using a HIOKI RM2610 to quantify both composite volume resistivity and electrode–collector interface resistance. Cross‐sectional samples were prepared by ion milling (CP‐8000 Plus, COXEM) to obtain artifact‐free surfaces. SEM (SU8600, Hitachi) was employed to observe microstructural changes before and after cycling. XPS (Thermo Fisher K‐Alpha^+^) was performed with binding energies calibrated to the C 1s peak at 284.8 eV, and data were processed using CasaXPS software. ToF‐SIMS (ION‐TOF M6, Münster, Germany) was performed in negative ion polarity mode to conduct depth profiling and image mapping. The analysis employed a Bi_3_^+^ primary ion beam (30 keV, 0.4 pA, raster size 100 µm × 100 µm) and sputtering with Cs^+^ ions (1 keV, 50 nA, raster size 300 µm × 300 µm).

### 4.4 Electrochemical Characterization

Half‐cell anode slurries were prepared with 98 wt.% µSi and 2 wt.% binder, based on a total solid content of 2 g. For PVDF‐based slurries, 1.37 g of NMP was added to achieve a solid loading of approximately 50 wt.%. In the case of IRCB, 590 µL of EDA was added to obtain a solid loading of ∼63.4 wt.%. All slurries were mixed using a planetary centrifugal mixer (Thinky ARE‐310). PVDF slurries were mixed three times for 10 min each, while IRCB slurries underwent a 10 min mixing step, thermal curing at 50 °C for 30 min, and two additional 10 min mixing steps. The slurries were then cast onto Cu foil using a doctor blade. Drying was carried out in a convection oven at 80 °C (25 min for PVDF, 5 min for IRCB), followed by vacuum drying at 80 °C for 6 h. No calendering was applied. The areal capacity of the half‐cell anodes was adjusted to 3.0 mAh cm^–^^2^.

Full cell anodes were prepared using the same procedure, but with an increased areal capacity of 4.4 mAh cm^–^^2^. Cathodes were fabricated by dry mixing NCM811, LPSCl, C65, and PTFE in a 70:27:2:1 weight ratio using a mortar and pestle. The powder mixture was then pressed into freestanding films and punched into circular disks. The cathode capacity was balanced to maintain an N/P ratio of 1.1.

All‐solid‐state cells were assembled in an Ar‐filled glovebox (H_2_O/O_2_< 0.1 ppm). For half cells, the anode and LPSCl were cold‐pressed at 394.7 MPa, after which a Li metal counter electrode was applied under 56.39 MPa. For full cells, the cathode, LPSCl, and anode were stacked and pressed together at 394.7 MPa. During electrochemical cycling, external pressure was maintained by applying a torque of 7–8 N·m. Galvanostatic cycling was conducted at 30 °C using a NEWARE battery tester. Formation cycles were carried out at 0.05C. Long‐term cycling of half cells was performed at 0.2C, while full cells were tested at both 0.2C and 1.0C depending on the protocol. EIS was measured using a BioLogic analyzer over a frequency range of 1 MHz to 0.1 Hz with an amplitude of 10 mV. In situ EIS was conducted during the first cycle at selected voltage steps. DRT analysis was performed using MATLAB to deconvolute frequency‐dependent resistance components [54]. Lithium‐ion diffusion coefficients were obtained via GITT, using 10 min current pulses followed by 1 h relaxation intervals. The diffusion coefficient *D^GITT^* was calculated according to the equation.

$$\;D^{GITT}=\frac{4}{\pi \tau }{\left(\frac{m_{B}}{\rho S}\right)}^{2}{\left(\frac{\Delta E_{s}}{\Delta E_{t}}\right)}^{2}$$

where *m_B_* is the mass of the active material (g), ρ is its density (g cm^–^^3^), *S* is the electrode–electrolyte contact area (cm^2^), τ is the duration of the current pulse (s), Δ*E_s_* is the steady‐state voltage change (V), and Δ*E_t_* ​ is the total voltage change during the current pulse (V).

### 4.5 Density Functional Theory Calculations

DFT calculations were conducted with the Vienna *ab‐initio* simulation package (VASP) [57]. The Perdew–Burke–Ernzerhof generalized gradient approximation (GGA‐PBE) was chosen for the exchange‐correlation functionals. Projector‐augmented‐wave potentials were used with valence configurations of 2*s* ^1^, 3*s* ^2^3*p* ^2^, 2*p* ^2^, 2*s* ^2^2*p* ^4^, 2*s* ^2^2*p* ^3^, 2*s* ^2^2*p* ^5^, and 1*s* ^1^ for Li, Si, C, O, N, F, and H, respectively. Plane waves with an energy cutoff of 400 eV were determined based on the convergence test within 0.01 eV/atom. A 4 × 4 × 1 mesh within the Monkhorst–Pack scheme [58]. was adopted for surface slabs, respectively. The threshold for convergence of electronic self‐consistent iterations was set to 10^−6^ eV/cell. The cell parameters and atomic positions were relaxed until the remaining force reached 1 × 10^−2^ eV/Å. A 6‐layer Si (111) slab was modeled with the bottom three layers fixed. The dimensions of the slab model were 15.46 × 15.46 × 29.47 Å^3^ (96 atoms). The slab was separated along the *z*‐axis by a vacuum region (∼ 15 Å). For modelling amorphous Li, Si atoms were randomly displaced within a range of –1 to 1 Å in all x, y, and z directions. For modelling amorphous Li_x_Si, eight Li atoms were inserted into the subsurface of the top Si layer, where space was available for Li atoms to occupy.

## Author Contributions

**Chanho Lee** contributed to writing – original draft, visualization, methodology, investigation, formal analysis, and conceptualization. **Yuri Nam** contributed to writing – original draft, visualization, methodology, investigation, and formal analysis. **Incheol Jeong** contributed to writing – original draft, methodology, investigation, and formal analysis. **Seo Eun Lee** contributed to the investigation and methodology. **Taewook Kim** contributed to the investigation and methodology. **Jinhyung Kim** contributed to the investigation. **Wooseup Jo** contributed to the investigation. **Moonsu Yoon** contributed to supervision, conceptualization, and resources. **Jongkyeong Lim** contributed to supervision, conceptualization, and resources. **Seho Sun** contributed to supervision, conceptualization, and resources. **Junghyun Choi** contributed to writing – review and editing, supervision, project administration, methodology, funding acquisition, and conceptualization. **Chan Ho Park** contributed to writing – review and editing, supervision, project administration, methodology, and conceptualization, and **Dongsoo Lee** contributed to writing – review and editing, supervision, project administration, methodology, funding acquisition, and conceptualization.

## Conflicts of Interest

The authors declare no conflicts of interest.

## Supporting information




**Supporting File**: advs74578‐sup‐0001‐SuppMat.docx.

## Data Availability

The data that support the findings of this study are available from the corresponding author upon reasonable request.
