# Supplementary material for: Interfacially Reinforced Crosslinked Binder with Structural Integrity for Stable Micro‐Sized Silicon Anodes in All‐solid‐state Batteries
Source: Adv Sci (Weinh). 2026 Mar 2;13(26):e00022. doi: 10.1002/advs.202600022 (PMC13159130; doi:10.1002/advs.202600022)
Supplement: Supplementary file 1 — Supporting File: advs74578‐sup‐0001‐SuppMat.docx. [file ADVS-13-e00022-s001.docx]

**Interfacially Reinforced Crosslinked Binder with Structural Integrity for Stable Micro-sized Silicon Anodes in All-solid-state Batteries**

*Chanho Lee^1,^*^†^*, Yuri Nam^1,^*^†^*, Incheol Jeong^2,^*^†^*, Seo Eun Lee^1^, Taewook Kim^1^, Jinhyung Kim^1^, Wooseup Jo^1^, Moonsu Yoon^1^,* *Jongkyeong Lim^3^, Seho Sun^4^, Junghyun Choi^1,^*, Chan Ho Park^1,^* and Dongsoo Lee^1,^**

^1^ School of Chemical, Biological and Battery Engineering, Gachon University, 1342 Seongnam-daero, Sujeong-gu, Seongnam 13120, Republic of Korea

^2^ Resources Utilization Research Center, KIGAM, Daejeon 34132, Republic of Korea

^3^ Department of Mechanical Engineering, Gachon University, 1342 Seongnam-daero, Sujeong-gu, Seongnam-si, Gyeonggi-do, 13120 Republic of Korea

^4^ School of Chemical Engineering, Yeungnam University, Gyeongsan, 38541, Republic of Korea

*Corresponding Author

E-mail: [junghchoi@gachon.ac.kr](mailto:junghchoi@gachon.ac.kr); chhopark@gachon.ac.kr; dslee9117@gachon.ac.kr

† These authors contributed equally to this work.

**Keywords:** binder, in situ crosslinking, hydrogen bonding, micro silicon anodes, all-solid-state batteries


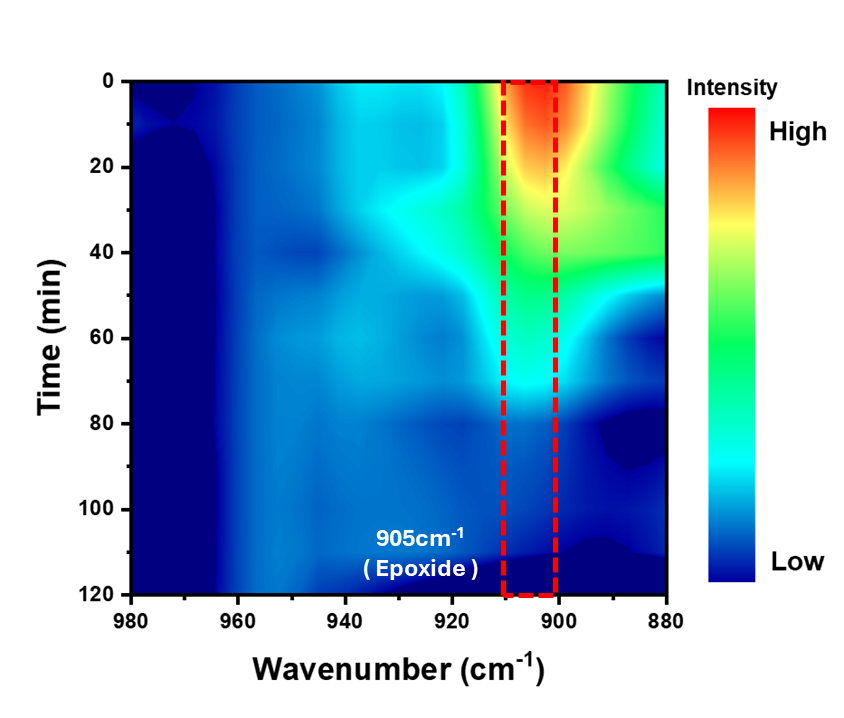


**Figure S1.** Time-resolved FT-IR spectra and contour map showing the consumption of epoxide groups for IRCB composites.


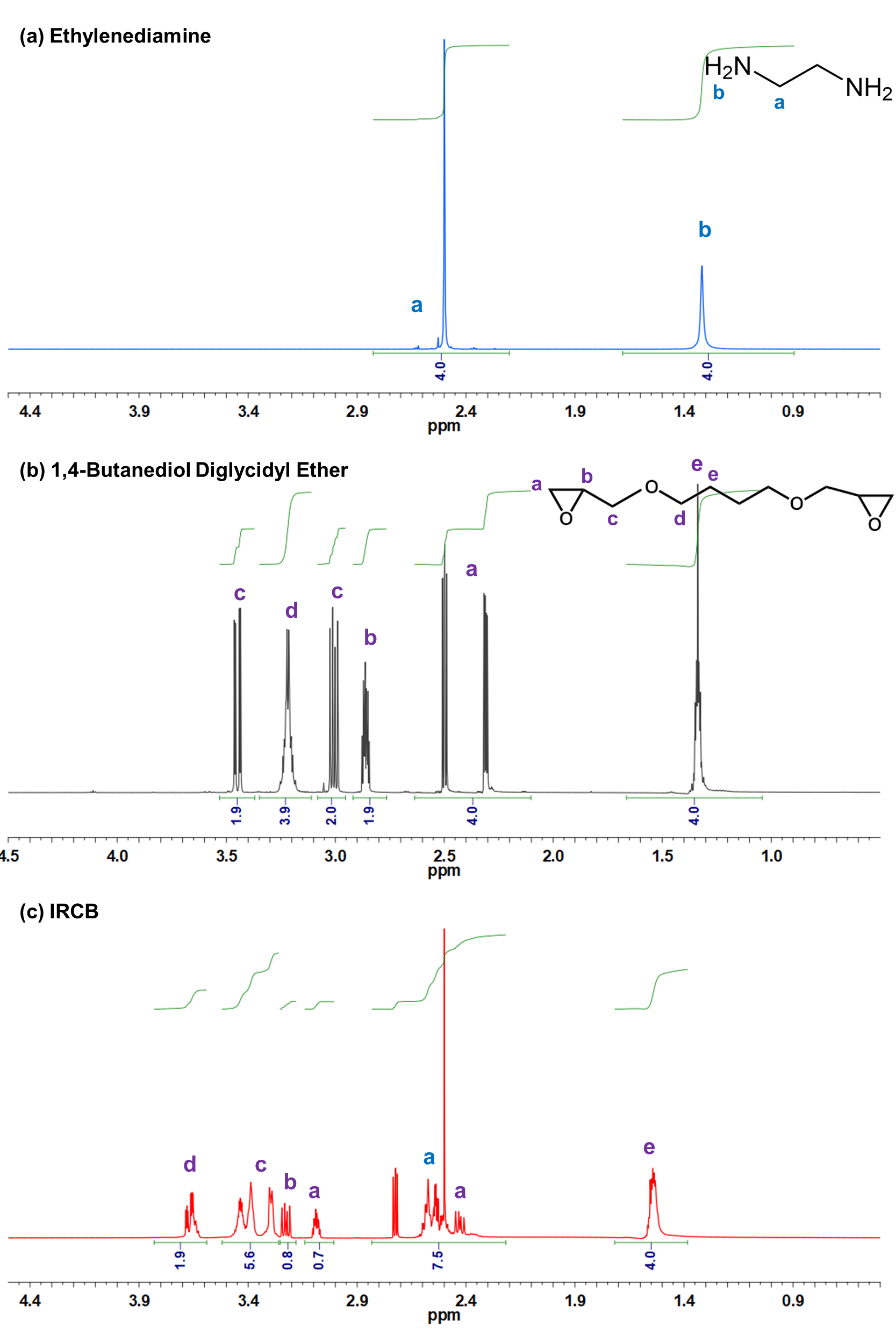


**Figure S2.** ^1^H NMR spectrum of (a) EDA, (b) BDDE, and (c) IRCB.


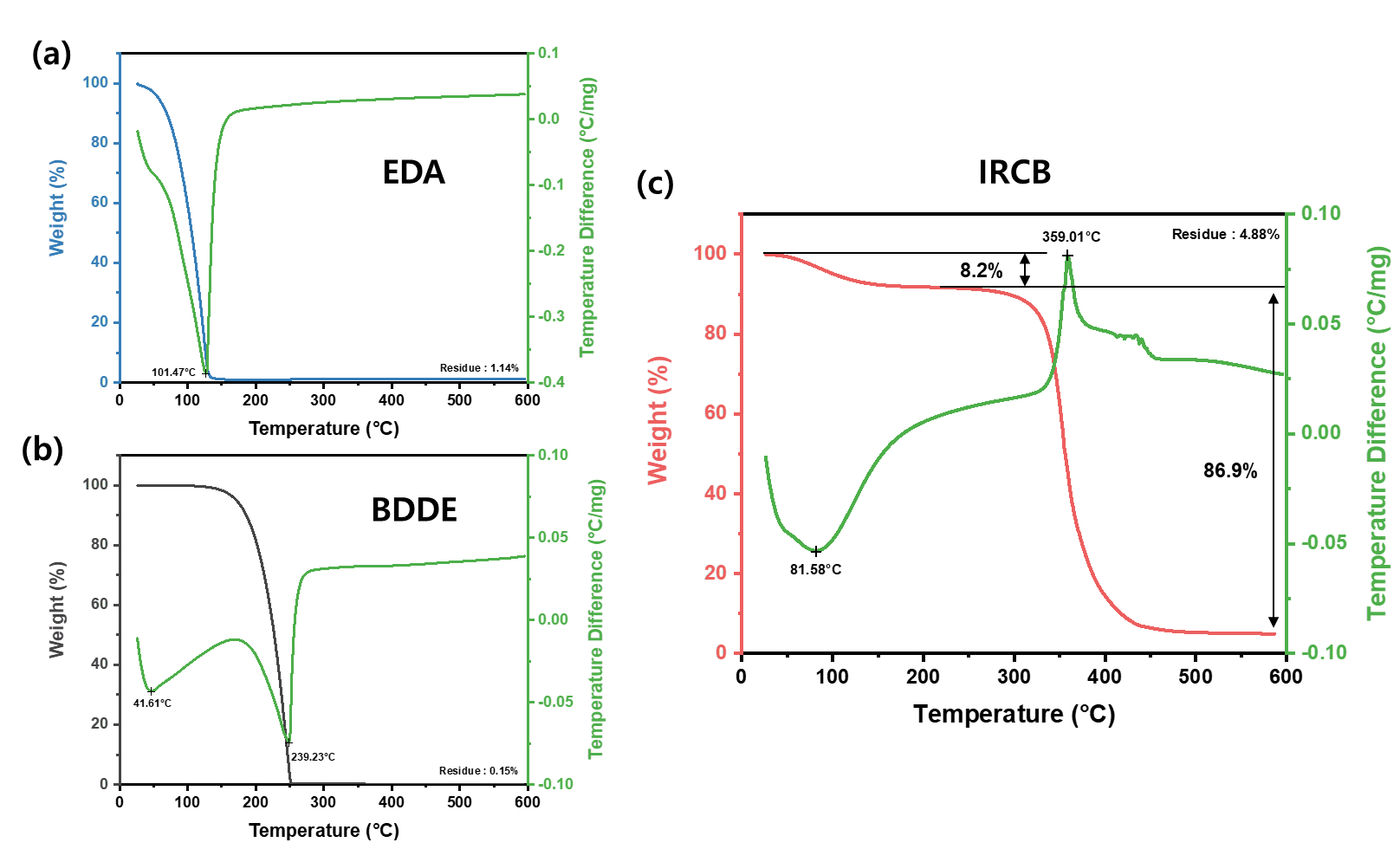


**Figure S3.** TGA analysis of (a) EDA, (b) BDDE, and (c) IRCB.


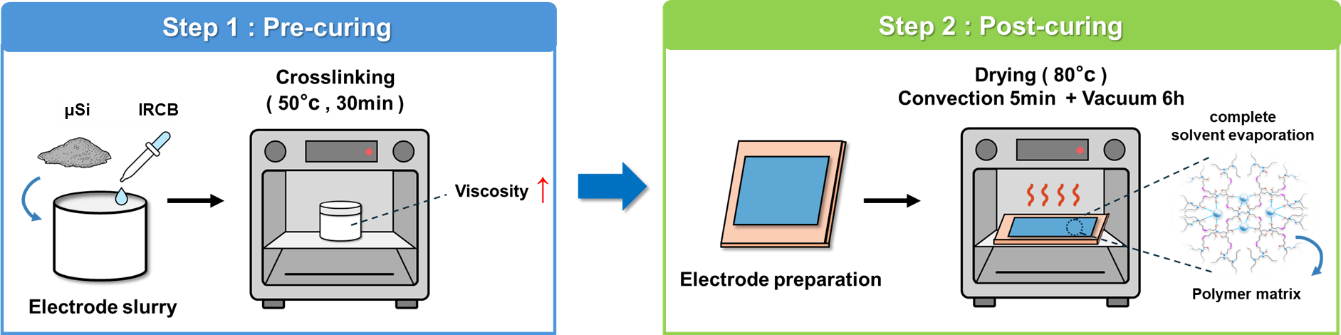


**Figure S4**. Schematic illustration of the two-step curing process designed to ensure uniform cross-linking throughout the IRCB binder matrix.


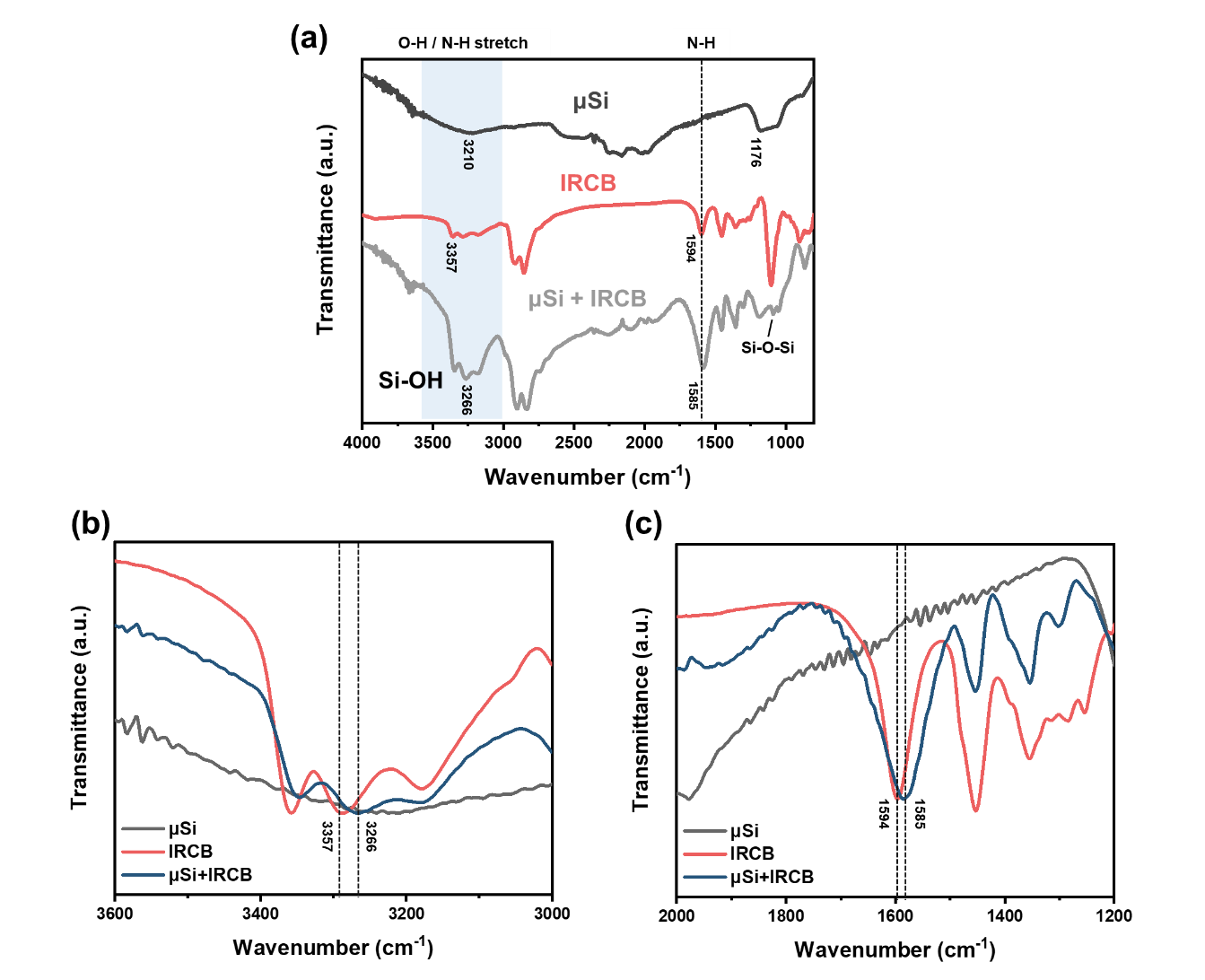


**Figure S5.** (a) FT-IR spectra of μSi, IRCB and μSi + IRCB composites. (b-c) Comparison of peak shift in normalized FT-IR spectra of μSi, IRCB and μSi + IRCB composites.

**Figure S6.** Residual solvent content of EDA and NMP over time under drying at 80 ^o^C, measured using an analytical balance. The mass of residual solvent was normalized to the initial slurry mass to obtain the relative evaporation ratio. Data are presented as mean ± standard deviation (n = 3). Both EDA and NMP exhibit gradual solvent loss at 80 ^o^C, with near-complete evaporation achieved within 2 hours. The inset image in panel a shows a representative dried film of EDA-based slurry (2 cm x 2 cm, 80 μm thickness) cast on Cu foil, demonstrating uniform drying behavior without crack formation.

**
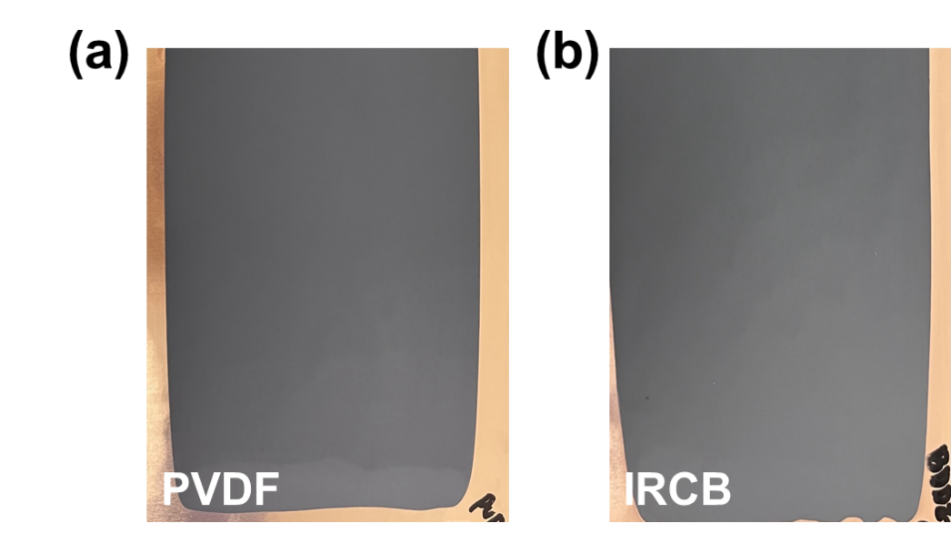
**

**Figure S7.** Digital photographs of dried μSi electrodes prepared with (a) PVDF and (b) IRCB binders, coated on Cu foil
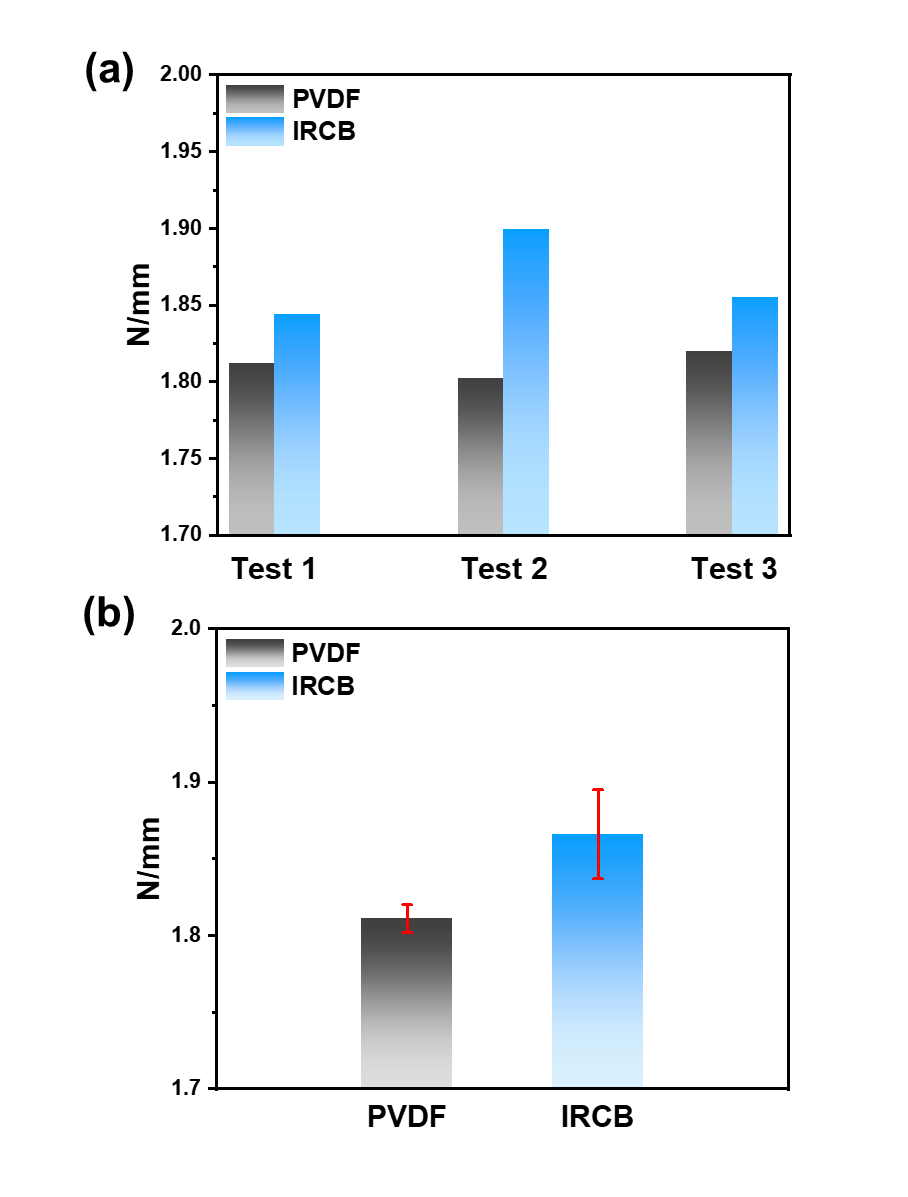


**Figure S8.** Interlayer shear force measurements from repeated SAICAS tests on μSi electrodes prepared with PVDF and IRCB binders. (a) Results from three consecutive measurements. (b) Averaged values from the three measurements.


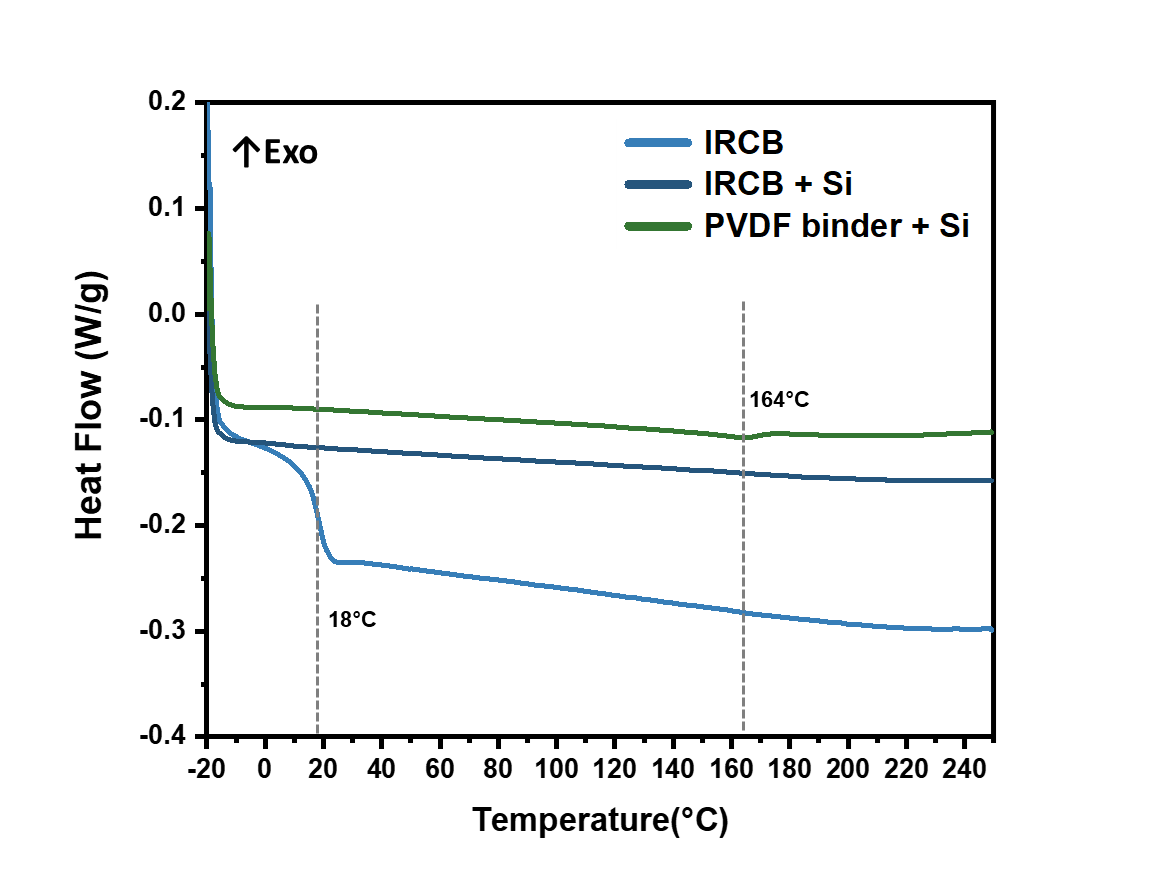


**Figure S9**. DSC second heating curves of IRCB without silicon particles (cyan line), IRCB with silicon particles (dark blue line), and PVDF with silicon particles as a reference (green line).

**Figure S10.** DMA analysis of the IRCB binder matrix along with the temperature from -20 to 200 °C (1 Hz; strain 0.05%; 5 °C/minute).


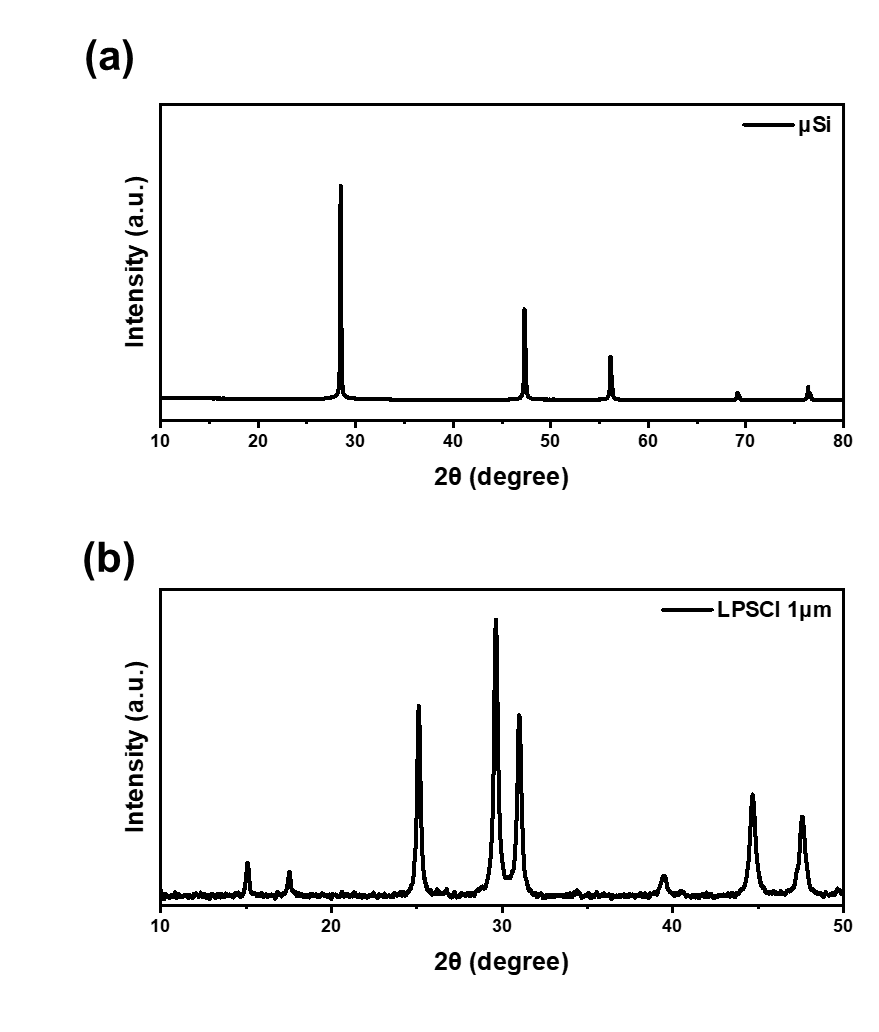


**Figure S11.** X-ray diffraction (XRD) patterns of (a) μSi and (b) LPSCl powder (average particle size ~1 μm) used in this study.

**
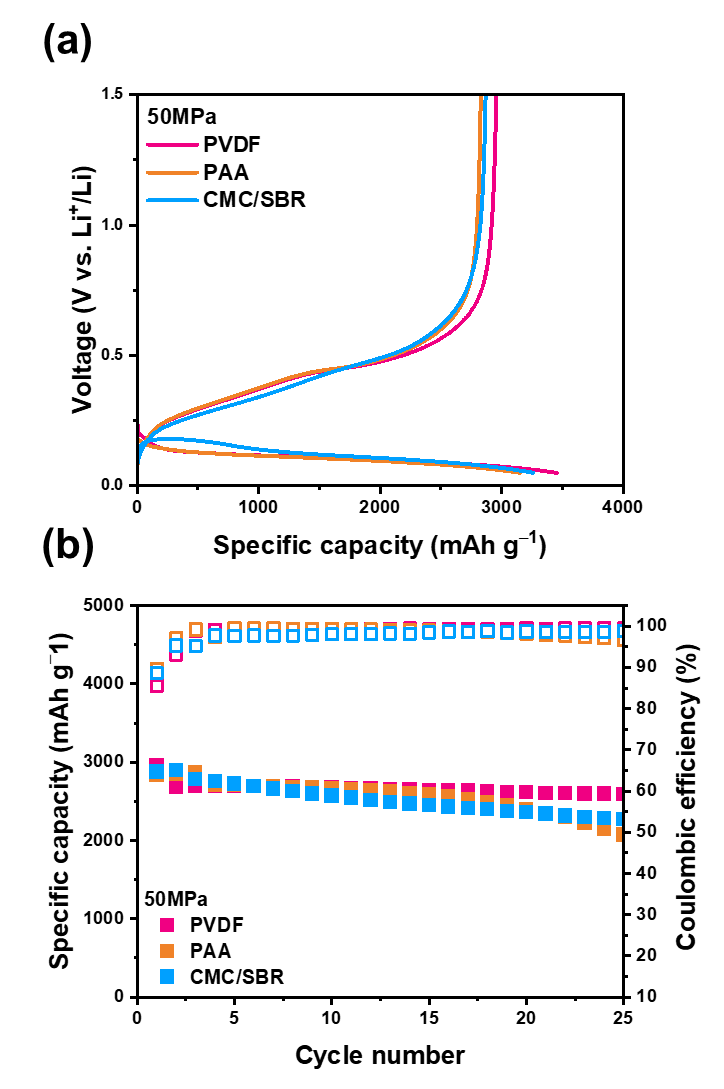
**

**Figure S12.** (a) Initial voltage profiles of μSi half cells employing PVDF, PAA and CMC/SBR binders. (b) Cycling performance and Coulombic efficiency over 25 cycles under the same conditions.

.
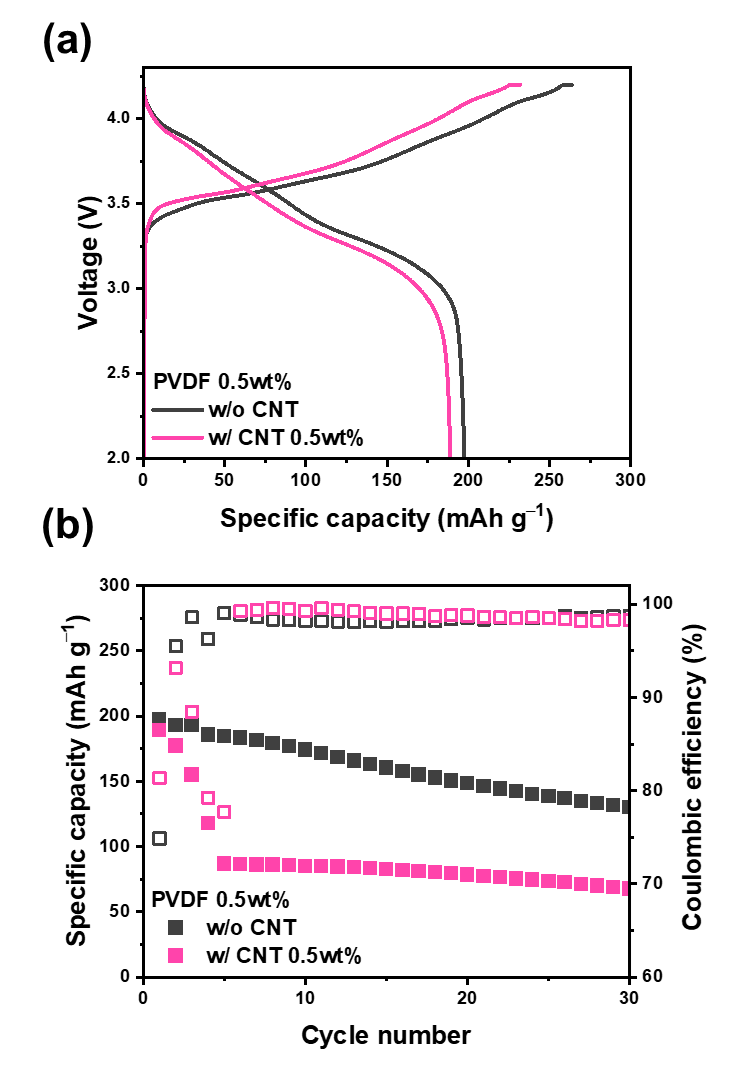


**Figure S13.** (a) Initial voltage profiles of PVDF-based cathodes (0.5 wt%) with and without 0.5 wt% CNT additive. (b) Cycling performance and Coulombic efficiency over 30 cycles, highlighting the adverse effects of CNT on interfacial stability.

**
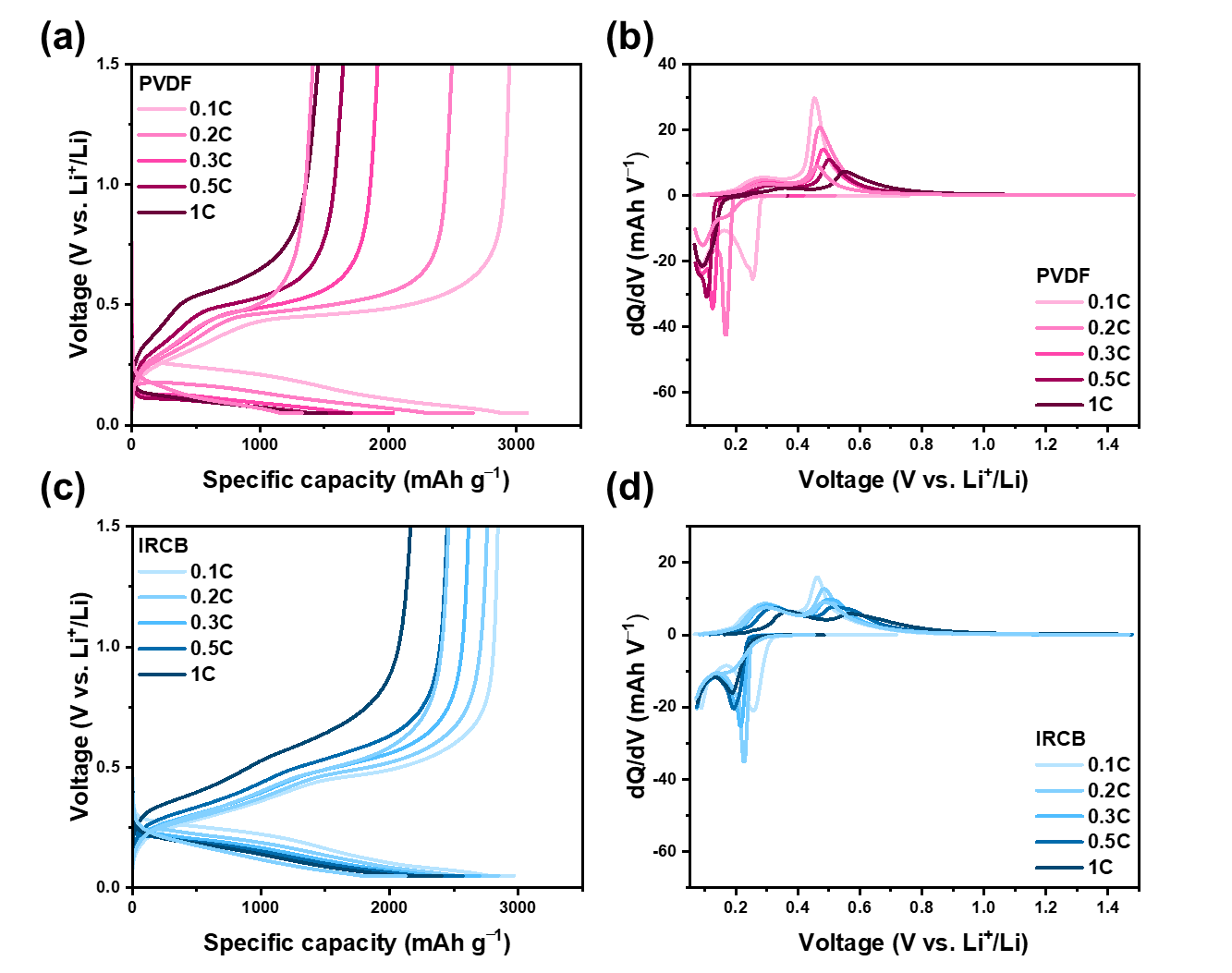
**

**Figure S14.** (a) Voltage profiles of PVDF electrodes during rate capability tests. (b) Corresponding dQ/dV plots for PVDF electrodes. (c) Voltage profiles of IRCB electrodes during rate capability tests. (d) Corresponding dQ/dV plots for IRCB electrodes.

**
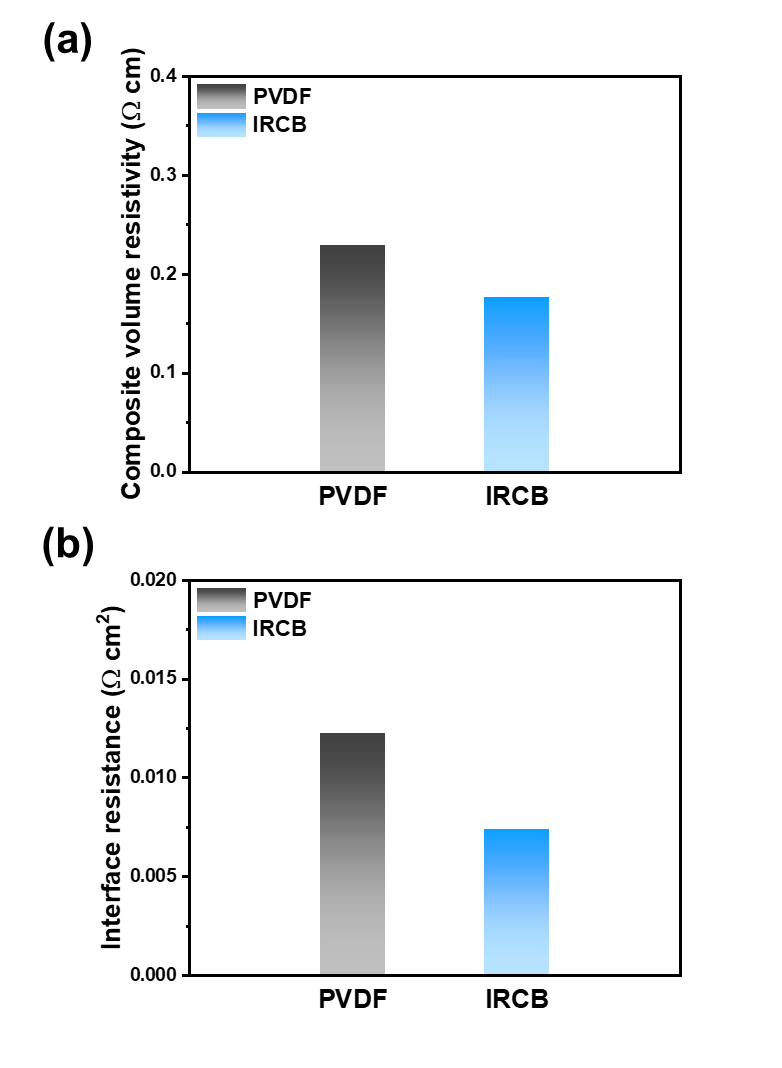
**

**Figure S15.** (a) Composite volume resistivity and (b) interface resistance of μSi electrodes containing PVDF or IRCB binders, measured using a HIOKI impedance analyzer.

**
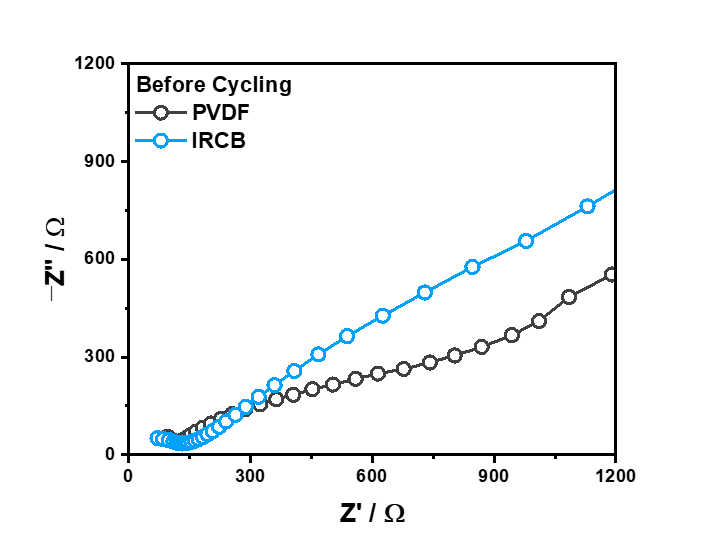
**

**Figure S16.** Electrochemical impedance spectra of half cells measured before cycling.

**
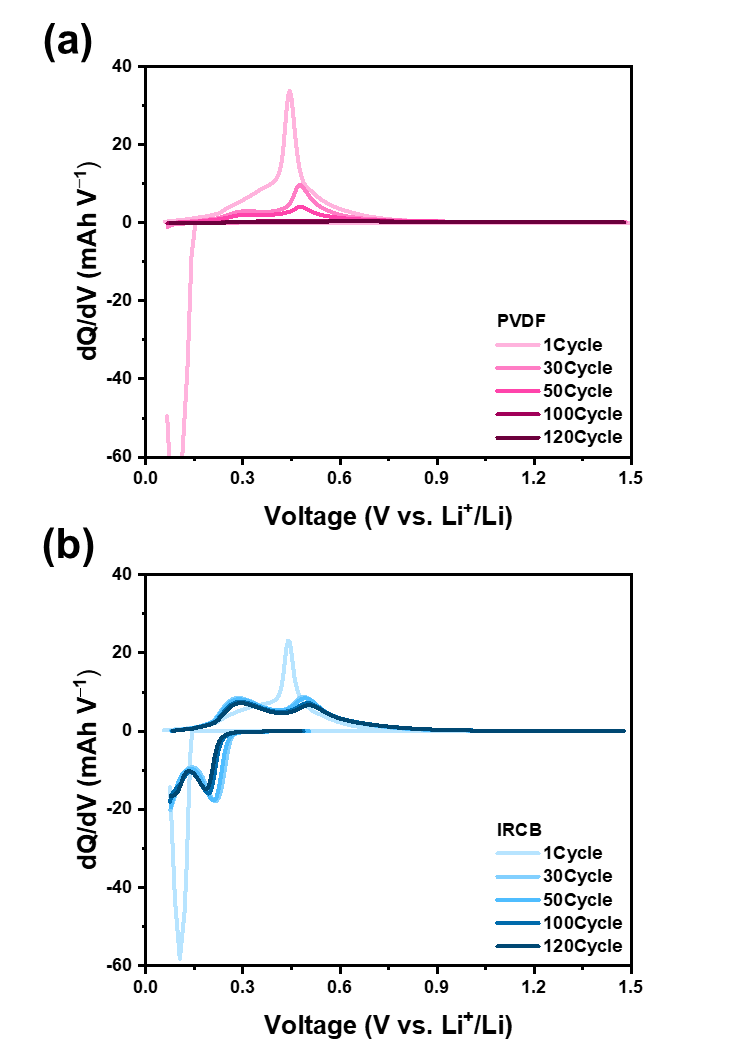
**

**Figure S17.** (a) dQ/dV plots of PVDF electrodes during cycling at 0.2 C. (b) dQ/dV plots of IRCB electrodes during cycling at 0.2 C.


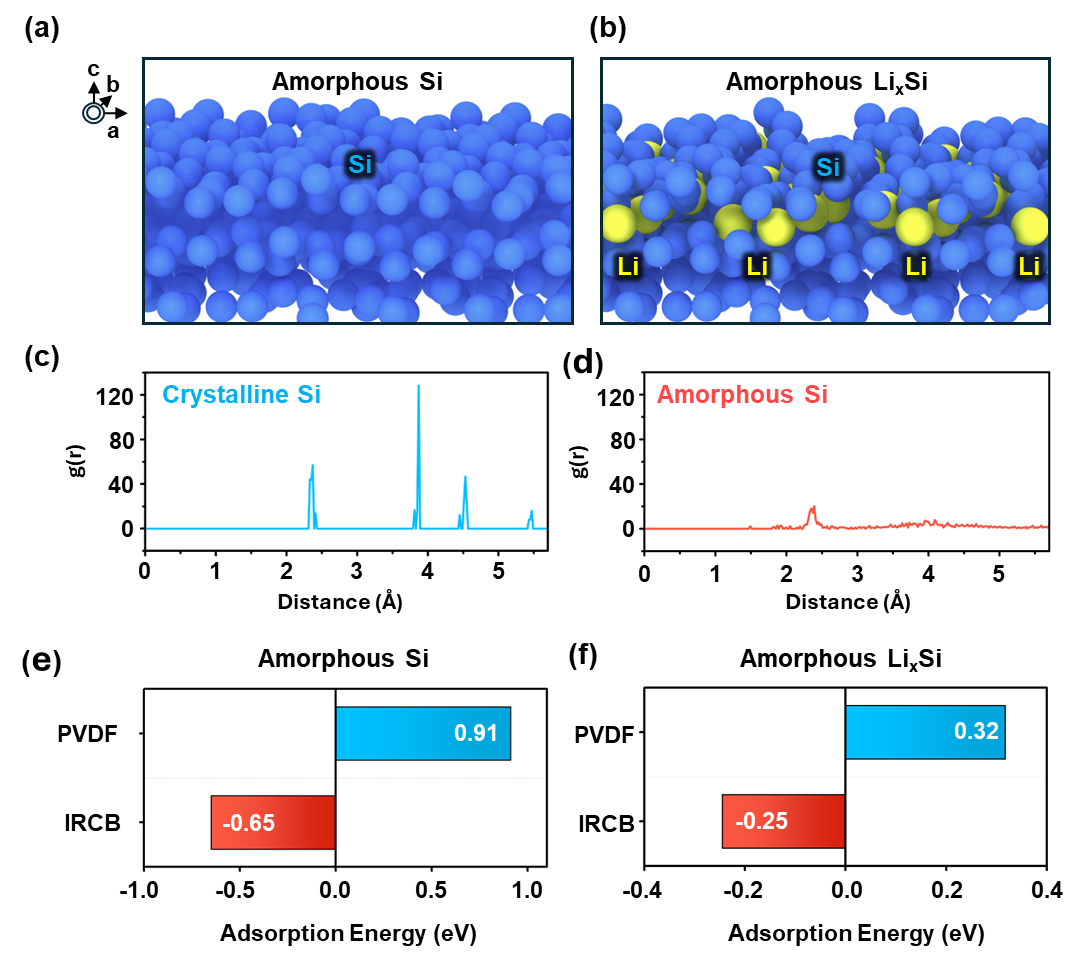


**Figure S18.** Structure model of (a) amorphous Si and (b) amorphous LixSi. Radial distribution function of (c) crystalline Si and (d) amorphous Si. Adsorption energies of PVDF and IRCB on (e) amorphous Si and (f) amorphous Li_x_Si.


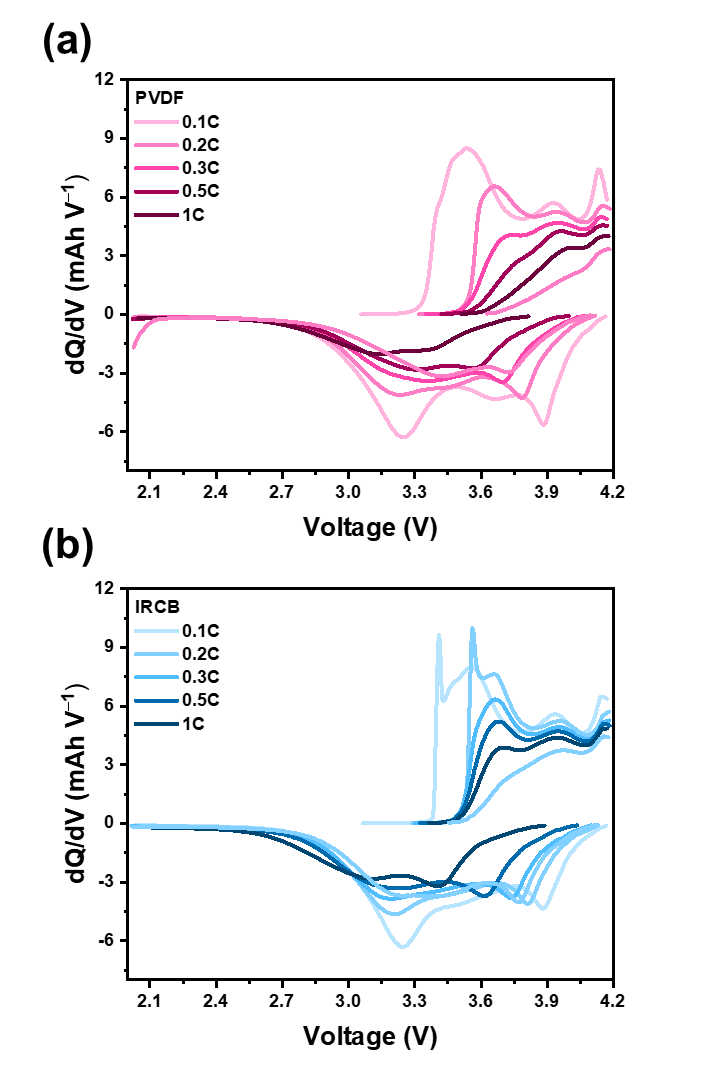


**Figure S19.** (a) dQ/dV plots of PVDF electrodes during rate performance testing. (b) dQ/dV plots of IRCB electrodes during rate performance testing.

**
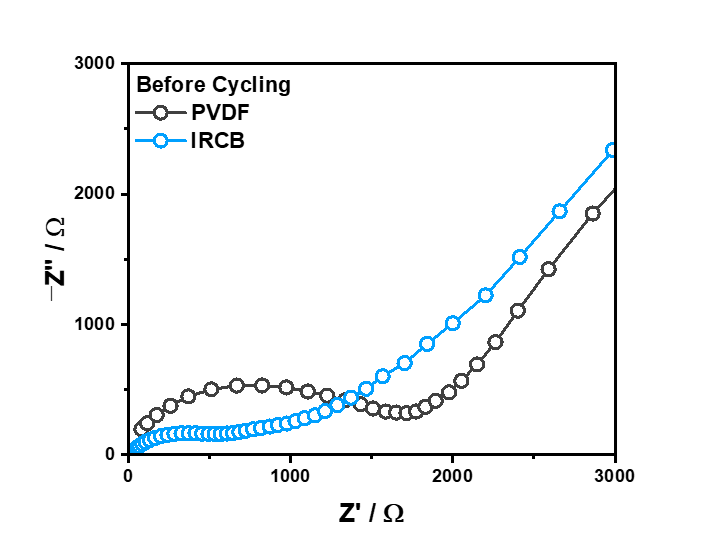
**

**Figure S20.** Electrochemical impedance spectra of full cells measured before cycling.

**
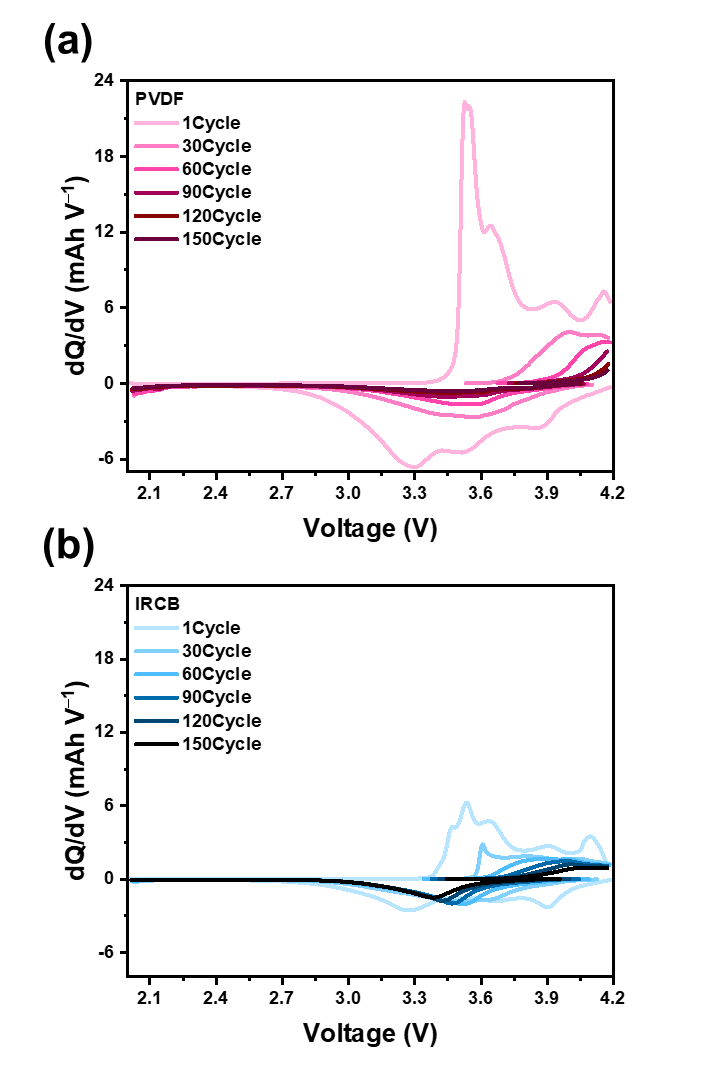
**

**Figure S21.** dQ/dV plots of full cells with PVDF and IRCB binders at 0.2 C (a) PVDF, (b) IRCB


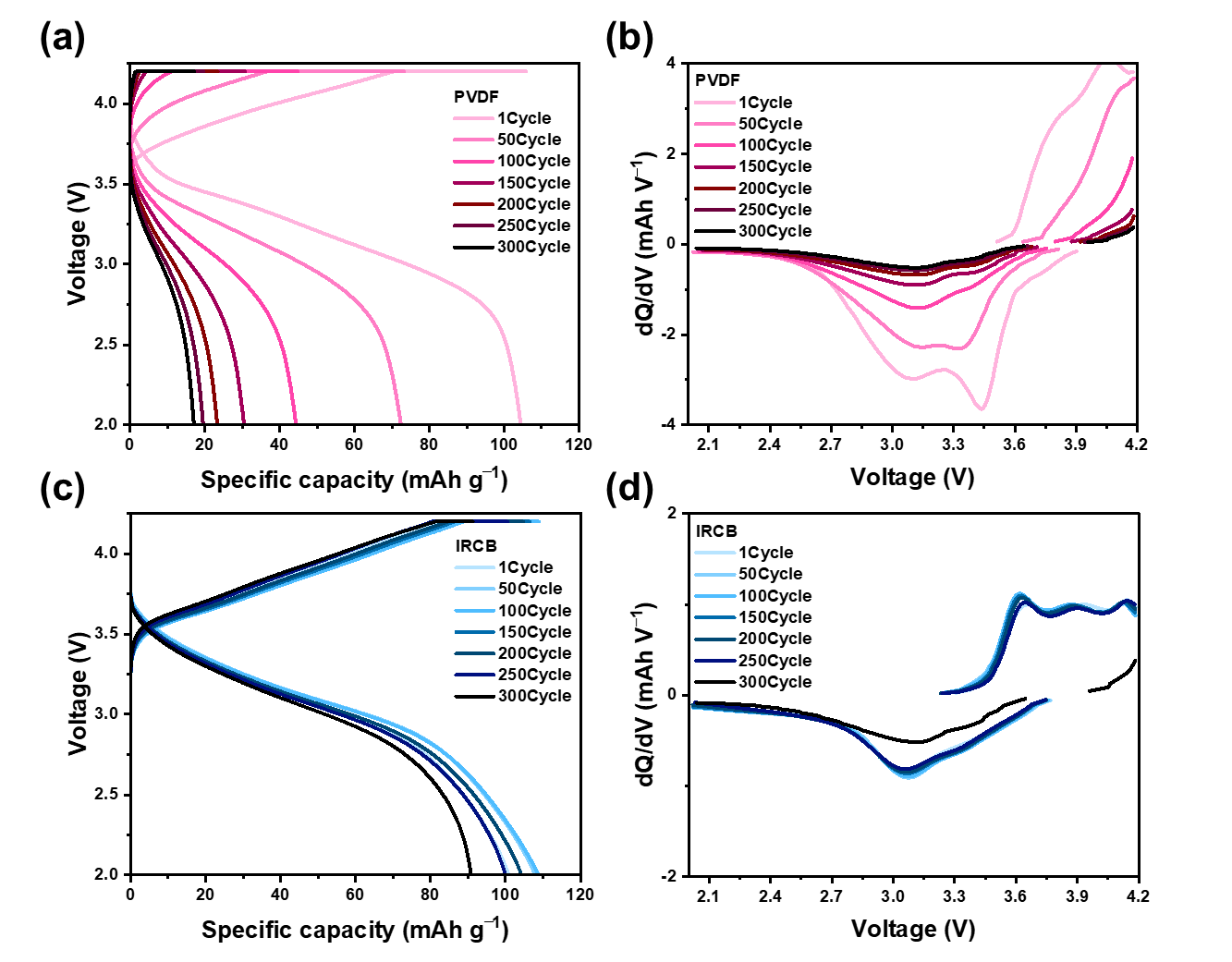


**Figure S22.** Full-cell 1 C performance (a,b) PVDF voltage profile and dQ/dV, (c,d) IRCB voltage profile and dQ/dV.


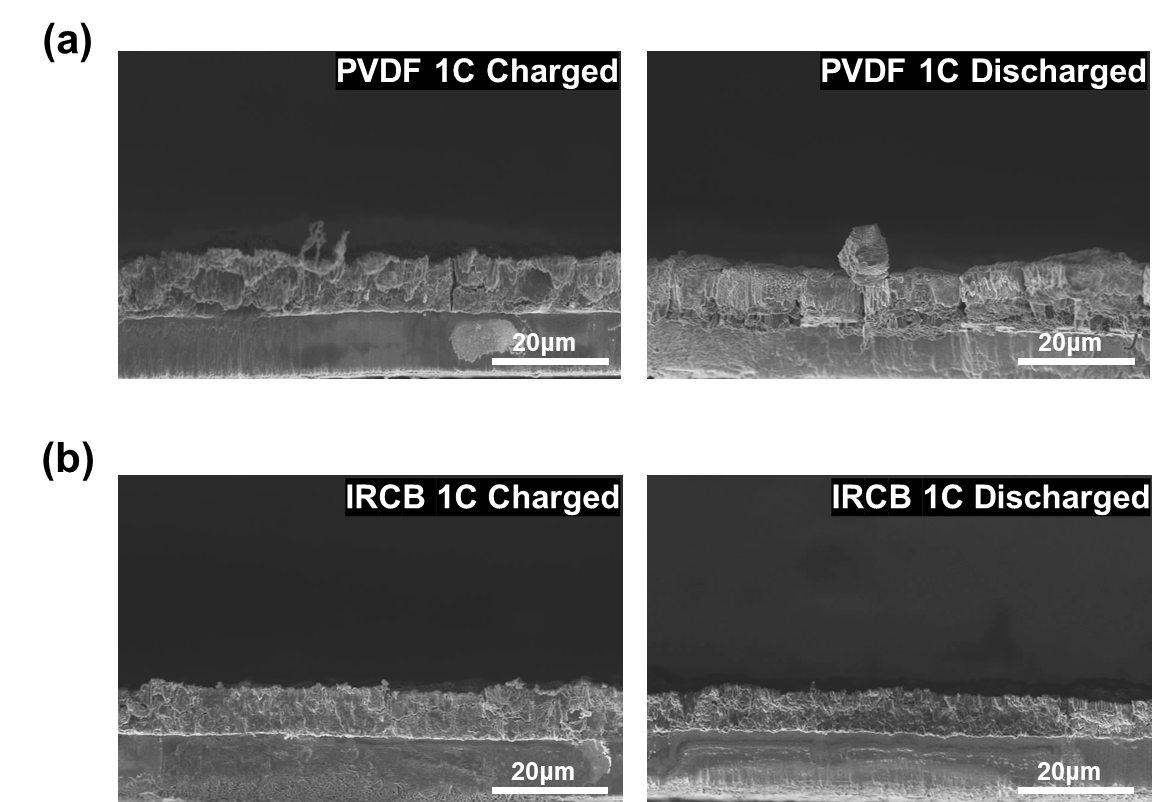


**Figure S23.** Cross-sectional SEM images of μSi anodes after cycling at 1 C (a) PVDF electrodes in charged and discharged states, and (b) IRCB electrodes in charged and discharged states.

**Figure S24.** Initial voltage profiles of full cells with PVDF and IRCB electrodes, measured under a reduced stack pressure of 10 MPa.


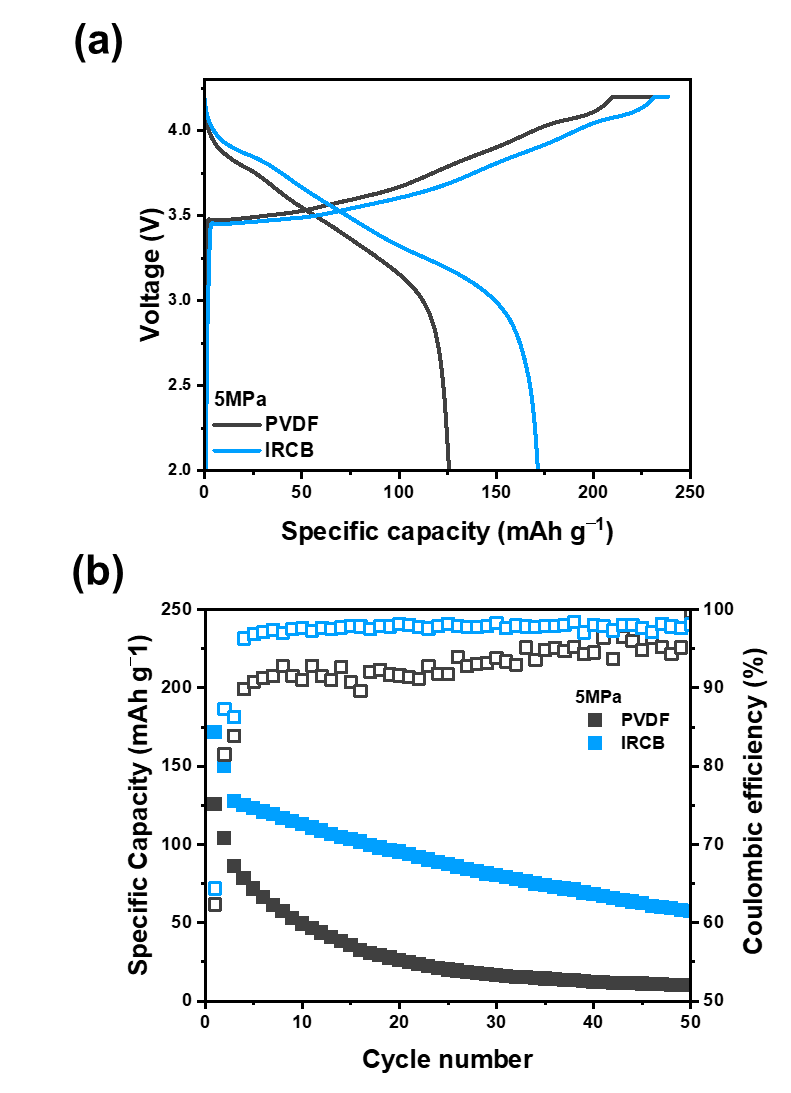


**Figure S25.** Electrochemical performance of μSi full cells operated under a reduced stack pressure of 5 MPa (a) initial charge–discharge voltage profiles of PVDF and IRCB electrodes, and (b) cycling performance and Coulombic efficiency at 0.2 C.


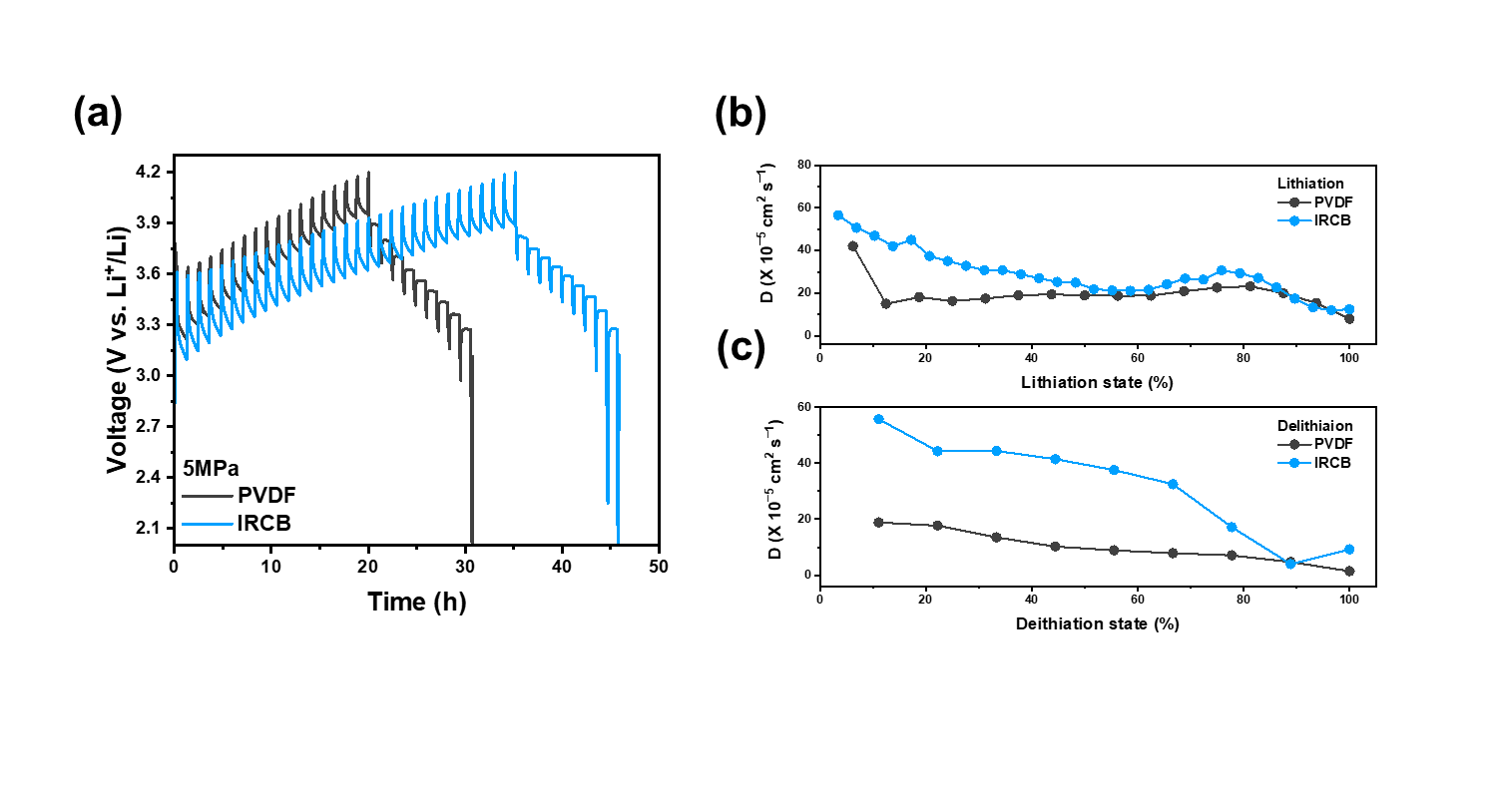


**Figure S26.** Lithium-ion diffusion analysis using GITT for μSi half cells with PVDF and IRCB electrodes under a reduced stack pressure of 5 MPa. (a) Voltage profiles under intermittent current application, (b) apparent Li^+^ diffusion coefficients during lithiation, and (c) apparent Li^+^ diffusion coefficients during delithiation.


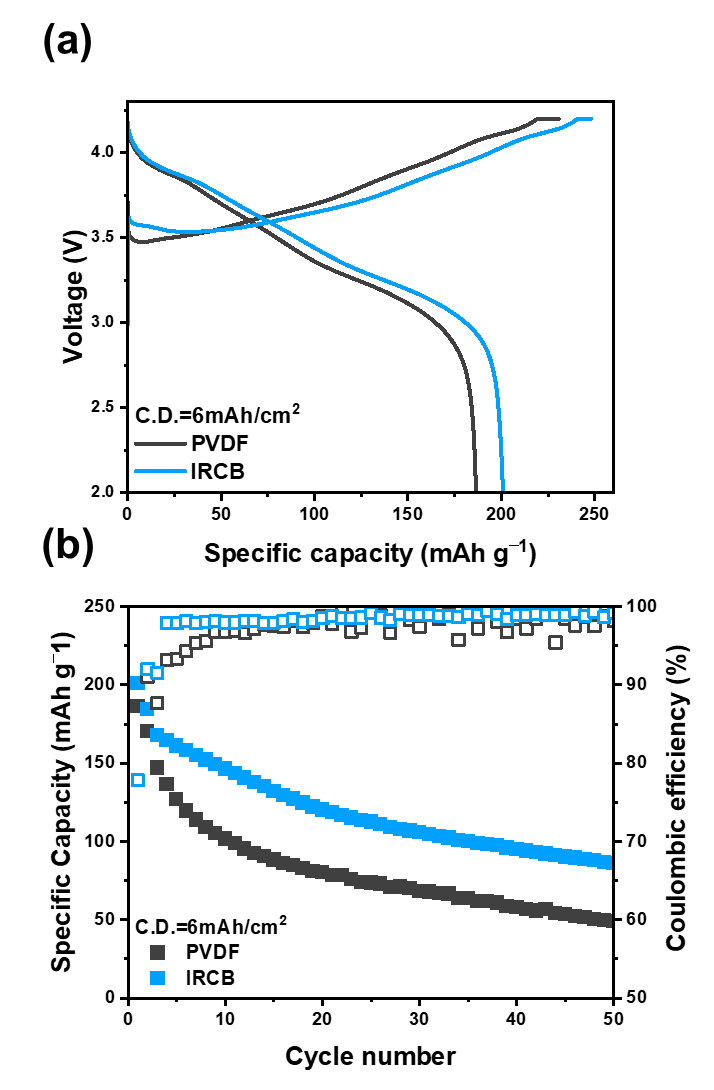


**Figure S27.** Electrochemical performance of high-loading cathodes (C.D. ≈ 6 mAh cm⁻²) evaluated under a stack pressure of **50 MPa** (a) initial charge–discharge profiles at 0.05 C, (b) cycling performance at 0.2 C.


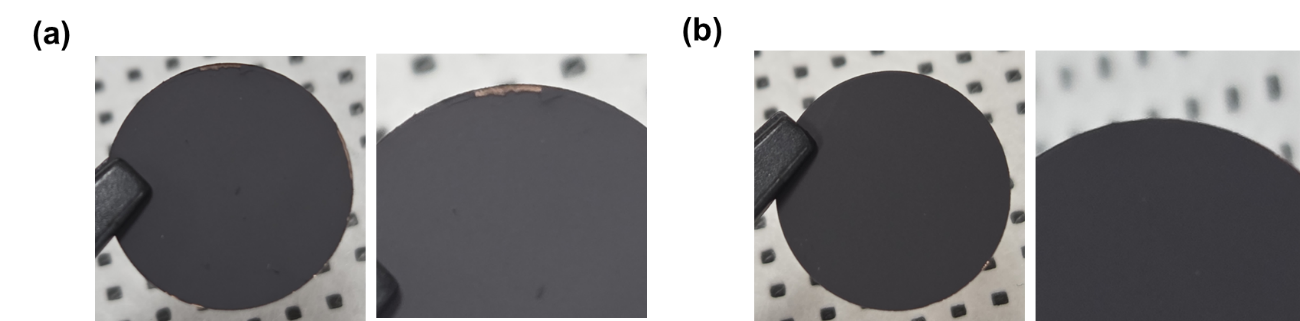


**Figure S28.** Photographs of high-loaded electrodes (C.D. ≈ 6.6 mAh cm^–2^) prepared with (a) PVDF and (b) IRCB binders.

**
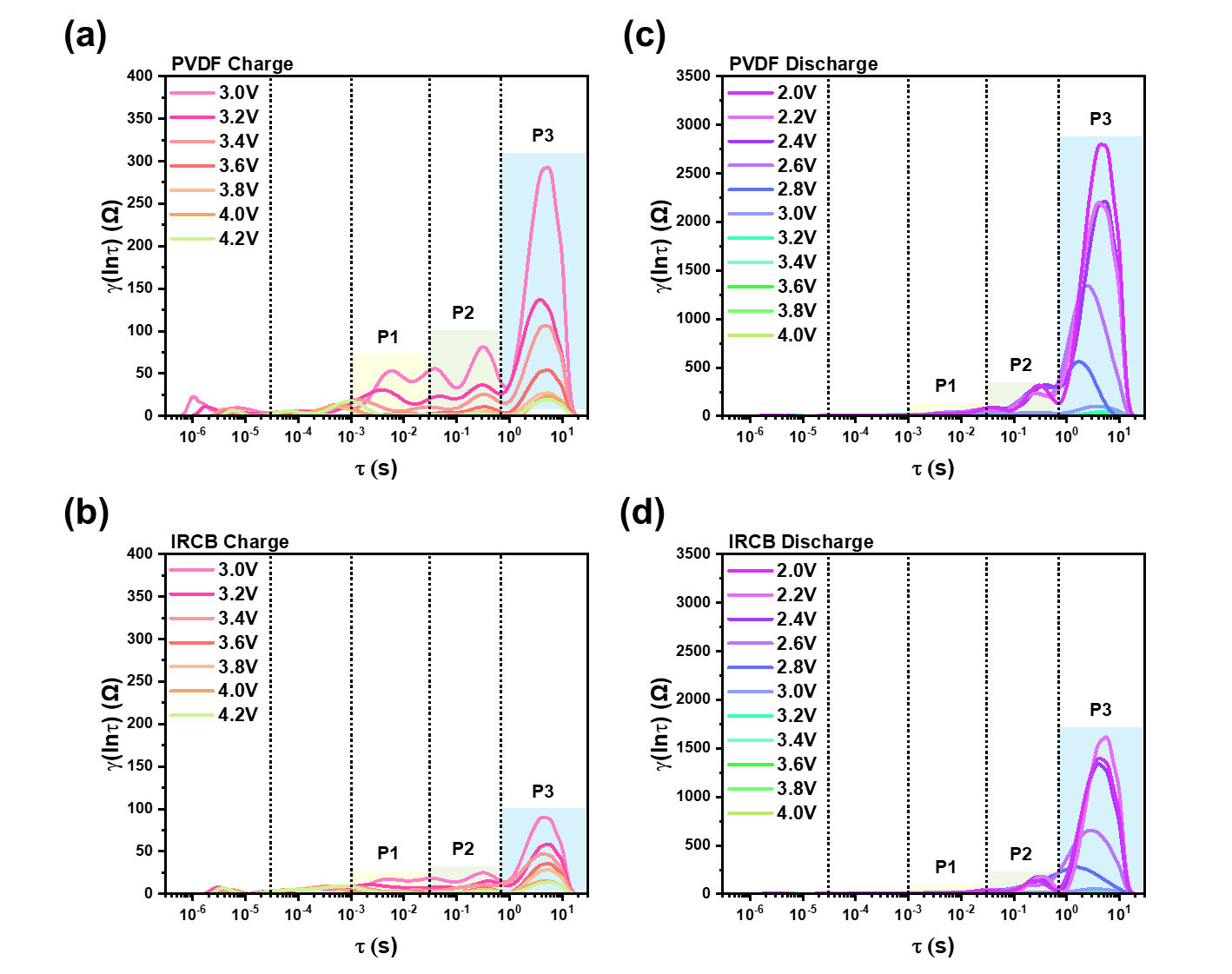
**

**Figure S29.** Distribution of relaxation time (DRT) spectra for full cells with PVDF and IRCB electrodes. (a, b) Spectra during charging and (c, d) during discharging.

**
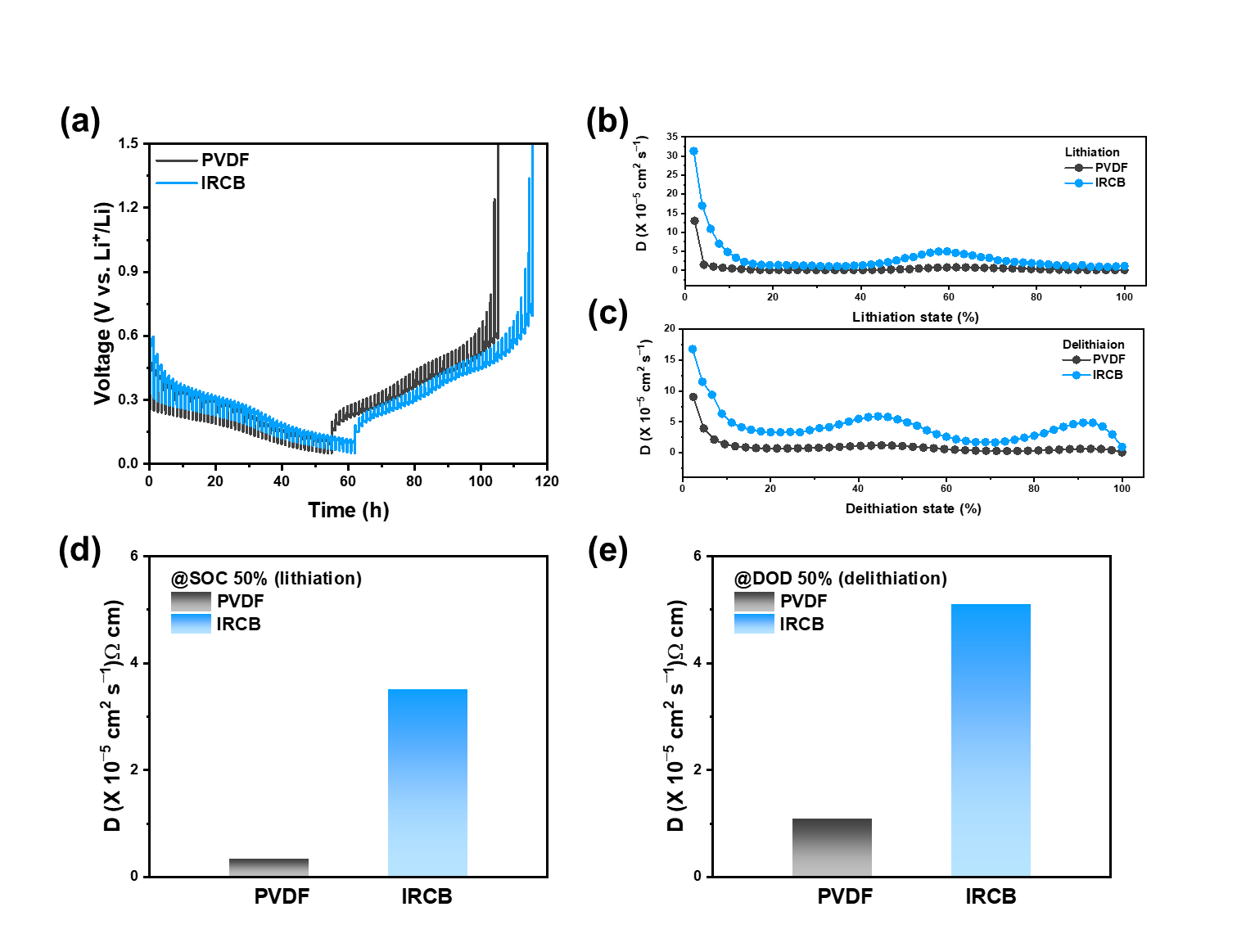
**

**Figure S30.** Lithium-ion diffusion analysis using GITT for μSi half-cells with PVDF and IRCB electrodes. (a) Voltage profiles under intermittent current application. (b) Diffusion coefficients during lithiation. (c) Diffusion coefficients during delithiation. (d) Diffusion coefficients at 50% SOC. (e) Diffusion coefficients at 50% DOD.

**
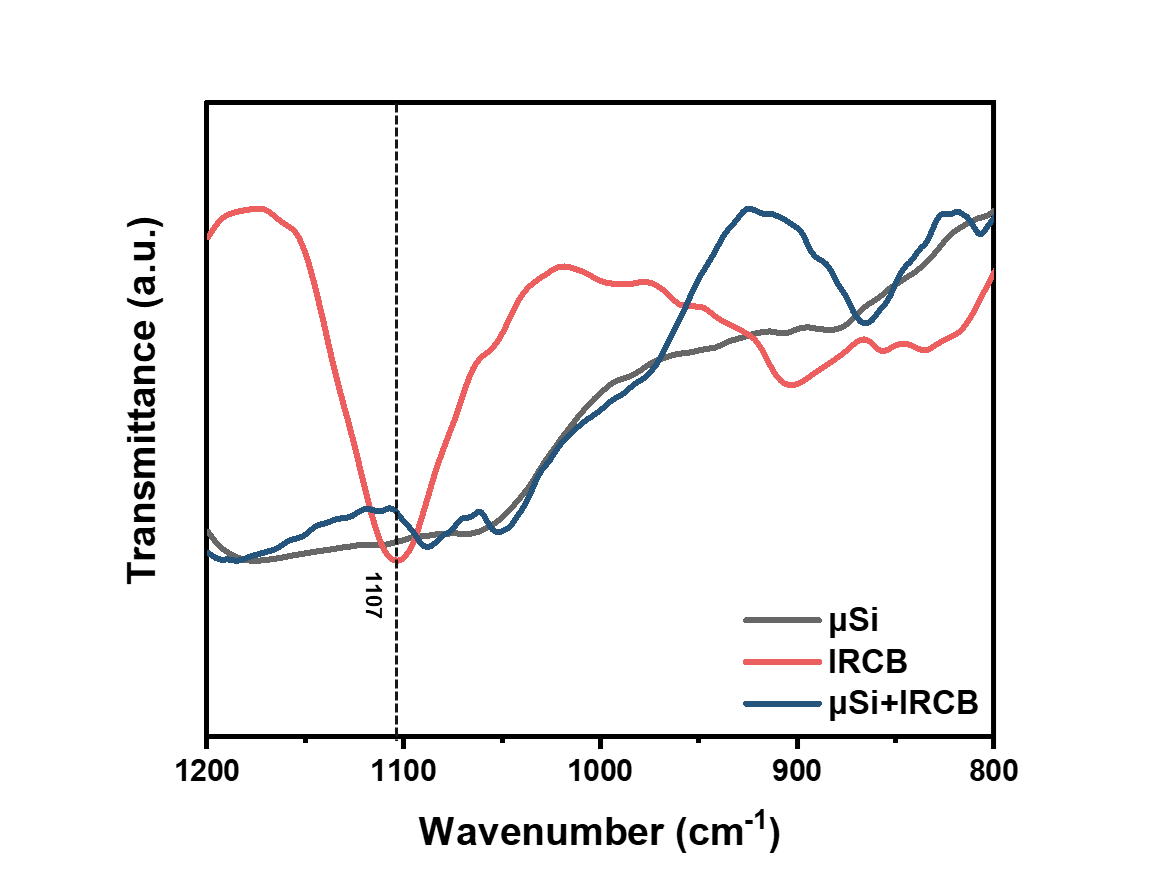
**

**Figure S31.** FT-IR spectra of μSi and μSi + IRCB composites.

**
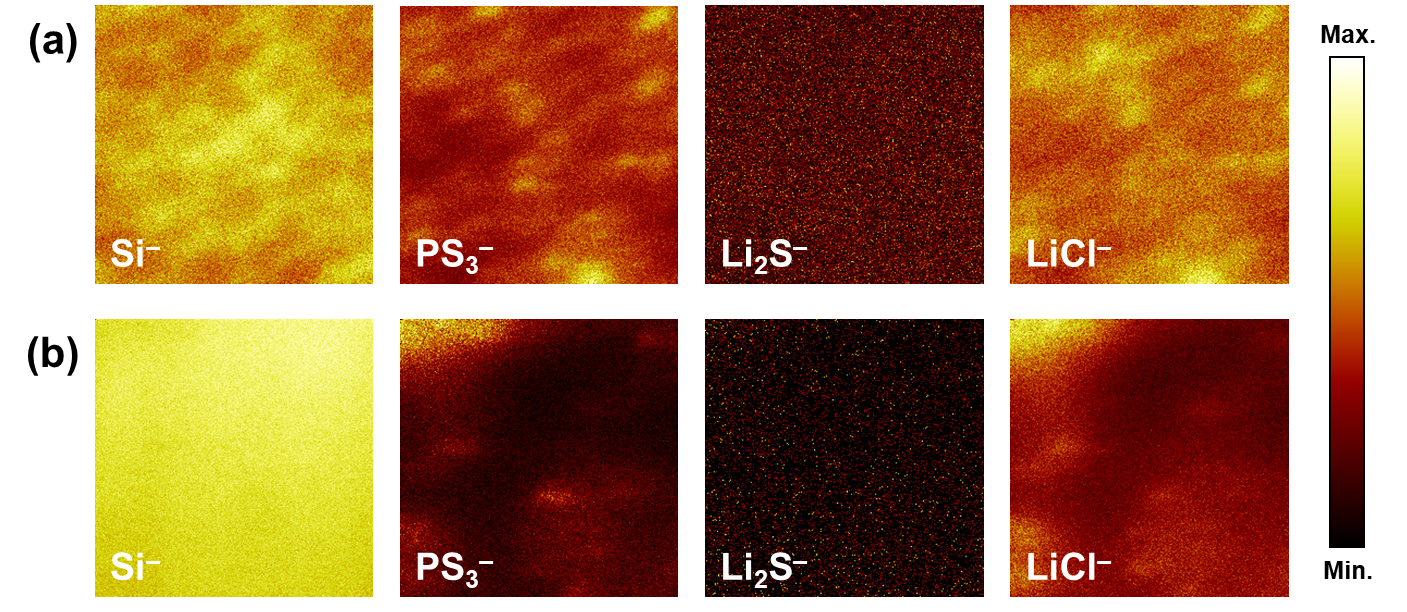
**

**Figure S32.** 2D ToF-SIMS images of Si^–^, PS_3_^–^, Li_2_S^–^, and LiCl_2_^–^ secondary ion distributions at the μSi/LPSCl interface after cycling (a) PVDF and (b) IRCB electrodes.

**Table S1.** Comparison of solvent evaporation behavior for EDA- and NMP-based slurries (80 μm thick on Cu foil) at 50 °C and 80 °C. The residual solvent mass was monitored over time to evaluate drying efficiency and film-forming stability.

| **Compound** | **Chemical Formula** | **Boiling Point (^o^C)** | **Vapor Pressure (V_p_) @25 ^o^C  (hPa)** | **Estimated V_p_ @ 50 ^o^C (hPa)** | **Estimated V_p_ @ 80 ^o^C (hPa)** | **Density  (g/cm^3^)** | **Volatility** |
| --- | --- | --- | --- | --- | --- | --- | --- |
| EDA | C_2_H_8_N_2_ | 116 | 12.1 | 49.3 | 204.5 | 0.9 | High |
| BDDE | C_10_H_18_O_4_ | 250 – 300 | 0.0005 | 0.005 | 0.06 | 1.1 | Low |
| NMP | C_5_H_9_NO | 202 | 0.3 | 1.8 | 10.1 | 1.03 | Low |

**Table S2.** Comparative physicochemical and toxicological properties of ethylenediamine (EDA) and N-methyl-2-pyrrolidone (NMP).

| **Category** | **Main production Route** | **CO2 Emissions (kg)** | **Total life cycle Emissions**  **(kg)** | **Regulatory Level** | **New plant Licensing** | **Occupational Exposure** |
| --- | --- | --- | --- | --- | --- | --- |
| Ethylenediamine | EDC + NH₃ reaction | No publicly available data  (under research for CO₂ capture) | Approximately  2.2–2.8kg  (cradle-to-gate) | Not listed | No mandatory carbon reduction report | ~10ppm |
| N-methyl-2-pyrrolidone | γ-Butyrolactone  + methylamine condensation | 3.72kg | 4.22kg | SVHC listed | Mandatory CO₂/LCA reporting | ~10ppm |

**Table S3.** Performance comparison of Si anodes in ASSB full cells under various current densities, including areal capacity, cycle number, capacity retention, stack pressure, and operating temperature.

| **Ref** | **Areal Capacity (mAh cm^-2^)** | **C-rate (C)** | **Current Density (mA cm^-2^)** | **Cycle number** | **Capacity Retention (%)** | **Stack pressure (MPa)** | **Temp. (˚C)** |
| --- | --- | --- | --- | --- | --- | --- | --- |
| This Work | 4.4 | 1 | 4.4 | 300 | 90 | 50 | 30 |
| R1^[1]^ | 5 | 1 | 5 | 500 | 80 | 50 | 25 |
| R2^[2]^ | 4.31 | 0.1 | 0.43 | 100 | 58 | 50 | 25 |
| R3^[3]^ | 2.54 | 1 | 2.54 | 1000 | 84 | 60 | 30 |
| R4^[4]^ | 0.16 | 0.33 | 0.05 | 1000 | 63 | 50 | 25 |
| R5^[5]^ | 2.76 | 0.07 | 0.19 | 47 | 79 | 75 | 25 |
| R6^[6]^ | 0.28 | 0.2 | 0.06 | 42 | 76 | 140 | 25 |
| R7^[7]^ | 1.95 | 0.4 | 0.78 | 100 | 82 | 20 | 25 |
| R8^[8]^ | 2.4 | 0.05 | 0.12 | 38 | 67 | 75 | 30 |
| R9^[9]^ | 2.67 | 0.5 | 1.335 | 300 | 57 | 120 | 25 |
| R10^[10]^ | 4 | 1.25 | 5 | 1000 | 74 | 75 | 25 |
| R11^[11]^ | 2.2 | 0.2 | 0.44 | 100 | 73 | 15 | 30 |
| R12^[12]^ | 2.2 | 0.5 | 1.1 | 100 | 72 | 5 | 30 |
| R13^[13]^ | 1.6 | 5 | 8 | 2000 | 60 | 75 | 25 |

[1] D. H. Tan, Y.-T. Chen, H. Yang*, et al.*, *Science* **2021**, 373, 1494.

[2] H. Huo, M. Jiang, Y. Bai*, et al.*, *Nature materials* **2024**, 23, 543.

[3] C. Li, Y. Wu, F. Ren*, et al.*, *Small* **2025**, 21, 2411451.

[4] D. Cao, X. Sun, Y. Li*, et al.*, *Advanced materials* **2022**, 34, 2200401.

[5] M. Yamamoto, Y. Terauchi, A. Sakuda, M. Takahashi, *Journal of Power Sources* **2018**, 402, 506.

[6] D. H. Kim, H. A. Lee, Y. B. Song*, et al.*, *Journal of Power Sources* **2019**, 426, 143.

[7] S. Cangaz, F. Hippauf, F. S. Reuter*, et al.*, *Advanced Energy Materials* **2020**, 10, 2001320.

[8] M. Yamamoto, Y. Terauchi, A. Sakuda, A. Kato, M. Takahashi, *Journal of Power Sources* **2020**, 473, 228595.

[9] Z. Fan, B. Ding, Z. Li*, et al.*, *Etransportation* **2023**, 18, 100277.

[10] S.-Y. Ham, E. Sebti, A. Cronk*, et al.*, *Nat Commun* **2024**, 15, 2991.

[11] S. Jun, G. Lee, Y. B. Song*, et al.*, *Small* **2024**, 20, 2309437.

[12] S. Jun, M. Jeong, B. Jang*, et al.*, *Nat Commun* **2025**.

[13] Z. Wang, X. Shen, S. Chen*, et al.*, *Advanced Materials* **2024**, 36, 2405025.
